# Supplementary material for: Draft Genome of Tanacetum Coccineum: Genomic Comparison of Closely Related Tanacetum-Family Plants
Source: Int J Mol Sci. 2022 Jun 24;23(13):7039. doi: 10.3390/ijms23137039 (PMC9267051; doi:10.3390/ijms23137039)
Supplement: Supplementary file 1 [file ijms-23-07039-s001.zip › ijms-1752027-supplementary.pdf]

Supplemental Figure S1.

```
TcoITS1           ----------------------CGAACCCTG-CAAA-GCAGAACGACCCGTGAACACGTAAAAACAACTGAGCGTCGAGTGGGT
TciITS1           ----------------------CGAACCCTG-CAAA-GCATAACGACCCGTGAACACGTAAAAACAACCGAGCGTCGAGTGGAT
AaITS1            ---------------------TCGAACCCTG-CAAA-GCAGAACGACCCGTGAACACGTAAAAACAACCGAGTGTCGATTGGAT
CsITS1            ---------------------TCGAACCCTG-CAAAAGCATAACGACCTATGAACACGTAAAAACAACCGAGTGTTTAGTGGAT
HaITS1            ---------------------TCGAACCCTG-CACA-GCAGAACGACCCGTGAACAAGTTAACACATCTGGCCTTGCCGGGACC
NtITS1            GTGAACTGCGGAAGGTCATTGTCGAAAC-TG-CAAG-GCAGAACGACC-GCGAACTTGTTTAAAAACCGGGGAGCGGTGCGGCC
AtITS1            ---------------------TCGATACCTGTCCAAAACAGAACGACCCGCGAACCAAAGATCACCACTCTCGGTGGGCCGGTT
                                        ***  * ** *     ** *******   ****        *   *            *

TcoITS1           TAAGCACTTGTTT-GATCCTCTTGATGCTTTGTCGATGTGCATT----TACTTGTG-----CTCTTTTGGACATG--GTGAATG
TciITS1           TAAGCACTTGCTT-GATCCTCTCGATGCTTTGTCGATGTGCATT----TACTTGTG-----TTCTTTTGGACACG--GTGAATG
AaITS1            CAAGCGCTTGTTT-GATCCTCTCGACGCTTTGTCGATGCGCATT----CGCTCGAG-----TTCTTTTGGACCTT--GTGAATG
CsITS1            TAAGCGCTTGTTT-GATCCTCTCGACGCTTTGTCGATGCACATT----TACTCGAG-----TCTTTTTGGACCTT--GTGAATG
HaITS1            GAAGCATTTGTTTCGGCCCTTGTGAGTCCTTGTCGACGTGCGTT----CATGCATGGACCATACCTTTGGTTTTGTCATGGATG
NtITS1            GGGGCGCTT-------------CGGC-CTCCGTCCGCGCGGATC----TCTCCCTA-----TCCCCGGGGCGCTCGTTCGCGTG
AtITS1            TCTTAGCCGATTCCTTGCCCGCCGGATCCGTGGTTTCGCGTATCGGCATGATCGGGAGCTTTTATCTCGGTCTTG--TCGTGCG
                                         *   *   *     *    *                         **         *   *

TcoITS1           TGTCATTGGCGCAA-TAACAA-CCCCCGGCACAATGTGTGCCAAGGAAAACTAAACTTAAGAAGGCTTGT-TTCATGTTGCCCC
TciITS1           CGTCATTGGCGCAA-TAACAA-CCCCCGGCACAATGCGTGCCAAGGAAAACTAAACTTAAGAAGGCTTGT-TTCGTGTTGCCCC
AaITS1            TGTCATTGGCGCAT-TAACAA-CCCCCGGCACAATGTGTGCCAAGGAAAACTAAACTCTAGAAGGCTCGTCTTCATGTTGCCCC
CsITS1            TGTCATTGGCTCAT-TAACAA-CCA--GGCACAATGTGTGCCAAGGAAAACTAAACTCAAGAAGGCTCGT-TTCATGTTGCCCC
HaITS1            TCATGTTGACAAAA-TAACAAACCCCCGGCACGAGATGTGCCAAGGAAAACCAAAATTAA-AGAACACGTGCTGTTGC-GCCCC
NtITS1            CGTGACGGGTG-AT-TAACGAACCCC-GGGGCGGAAAGCGCCAAGGAATACTAAATT---GAAAGCCTGC-CTCTCGC-GCCCC
AtITS1            CGTTGCTTCCGGATATCACAAAACCCCGGCACGAAAAGTGTCAAGGAACATGCAAAC--GAACGGCTGGCATTCGCCT--CCCC
                              *  * ** *  *   **  *     * * ******* *   **      *   *  *   *       ****

TcoITS1           -GTTCGCGGTGTGCTCATGGGACGTG-GCTTCTTTATAA-----------------TCAATCGCG--TCGCCCCTAACAAATCT
TciITS1           -GTTCGCGGTGTGCTCATGGGACGTG-GCTTCTTTATAA-----------------TCAATCGCG--TCGCCCCCAACAAATCT
AaITS1            CGTTCGCGGTGTGCTCATGGGATGTG-GCTTCTTTATAAATCGCG--------TCGCCCCCCACAATTCTCTGTAAAGGGAACT
CsITS1            TATTCGCGGTGTGCTCATGGGATGTG-GCTTCTTTATAA--------------TCCCCCCCCACAATTCTCCGTAAAGGGAACT
HaITS1            -GTTCGCGGTGTGCGCGCTGTTCGTG-GCTTCTTTGTAAACTTAAAACATCACGTCGCCCCCACCAGGCATCCCCTATAGGGCT
NtITS1            -GTCCGCGGTGCGCGCGGGGGACTTGTGCTTCTTTTGAAACACGCGTCGCCCCCCGCACTCCGCG-CCCAGAATCATGGACGCG
AtITS1            -GGAGACGGAGTGTGGGCGGATGCTGTGCTGCGAACTGAA---------------GTCTATCGTCGTCCCTCACCATC----CT
                        *** * *      *    ** *** *      *                      *      *             *

TcoITS1           T---TGT-TGGGAGCGGATATTGGTCTCCCGTGC-TCATGG----TGTG-GTTGGCCAAAATAGGAGTCCCTTCG--ATGGA-C
TciITS1           T---TGT-TGGGGGCGGATATTGGTCTCCCGTGC-TCATGG----TGTG-GTTGGCCAAAACAGGAGTACCTTCG--ATGGA-C
AaITS1            CGTGTTT-TGGGGGCGGATATTGGTCTCCCGTGC-TCACGG----CGTG-GTTGGCCGAAATAGGAGTCCCTTCG--ATGGA-C
CsITS1            TATGTTT-TGGGGGCGGATATTGGTCTCCCGTGC-TCATGG----CGTG-GTTGGCTAAAATAGGAGTCCCTTTG--ATGGA-T
HaITS1            GTCTTGTGTTGGGGCGGAGATTGGTCTCCCGTGC-CCATGG----CGTG-GTTGGCCTAAATAGGAGTCTCCTCGCGAGGGA-C
NtITS1            GTGGTGTCGCGGGACGGATACTGGCCTCCCGTGC-GCCCCGAGCATGCG-GTTGGCCTAAATGCGAGT-CCACGGCGACGGA-C
AtITS1            TTGCTGATGCGGGACGGAAGCTGGTCTCCCGTGTGTTACCG----CACGCGTTGGCCTAAATCCGAGCCAAGGACGCCTGGAGC
                      *     **  ****   *** ********       *       * ******  ***   ***            ***

TcoITS1           GCACGAACTAGTGGTGGTCGTAAA--AACCCTCGTTCTTTGTTCTGTGT---TAGTCGTAAGGAAAAACTCT-TCAAATAACCC
TciITS1           GCACGAACTAGTGGTGGTCGTAAA--AACCCTCGTTCTTTGTTCTGTGC---TAGTCGCAAGGAAAAACTCT-TCAAATAACCC
AaITS1            GCACGAACTAGTGGTGGTCGTAAA--AACCCTCGTCTTTTGTTTCGTGCCGTTAGTCGCAAGGGAAA-CTCT-AAGAA-AACCC
CsITS1            GCATAAACTAGTGGTGGTCGGAAA--AACCCTCGTCTTTTGTTTCGTGTTGATAGTCGCAAGGGAAA-CTCT-TTAAA-AACCC
HaITS1            GCACGG-CTAGTGGTGGTTGATAA--GACAGTCGTCTCGTGTCGTGCGTTTACTTTCTTGAGAGTAGATGCTCTTAAAGTACCC
NtITS1            GTCACGACAAGTGGTGGTTGAAACTCAACTCTCGTAATGTCGCGGCTCCGACCCGTCGCACGTTTGGGCTCC-----ACGACCC
AtITS1            GTACCGACATGCGGTGGT-GAACTTGATCCATTACATTTTATCGGTCGC------TCTTGTCCGGAAGCTGT---AGATGACCC
                  *      *  * ****** *        *  *       *               **                   *  ****

TcoITS1           -AATGTGTTGTCTTAGGATGACGCTTCGACC------------------------------
TciITS1           CAATGTGTTGTCTTAGGATGACGCTTCGACC------------------------------
AaITS1            CAACGTGTCGTCTTTTGACGGCGCTTCGA--------------------------------
CsITS1            CAATGTGTC----TTTGACGACGCTTCGA--------------------------------
HaITS1            TGATGTGTTGTCTTATGACGATGCTTCGATCGCGACCCCAGGTCAGGCGGGACTA------
NtITS1            T----TGTTGCGCTTAGA---CGCTCCGACCGCGACGCCAGTCAGCGGGACTACCCTGAGT
AtITS1            AAAGTCCATATA-------------------------------------------------
```

Supplemental Figure S1.

```
TcoITS2          ATCGCGTCGCCCCTAACAAATCTTTGT-----------------TGG-------GAGCGGATATTGGTCTCCCGTGC-TCATGG
TciITS2          ATCGCGTCGCCCCCAACAAATCTTTGT-----------------TGG-------GGGCGGATATTGGTCTCCCGTGC-TCATGG
AaITS2           ATCGCGTCGCCCCCCACAATTCTCTGTAAAGGGAACTCGTGTTTTGG-------GGGCGGATATTGGTCTCCCGTGC-TCACGG
CsITS2           -----ATCCCCCCCCACAATTCTCCGTAAAGGGAACTTATGTTTTGG-------GGGCGGATATTGGTCTCCCGTGC-TCATGG
HaITS2           ATCACGTCGCCCCCACCAGGCATCCCCTATAGGGCTGTCTTGTGTTG-------GGGCGGAGATTGGTCTCCCGTGC-CCATGG
NtITS2           ---GCGTCGCCCCCCGCACTCCGCGCCCAGAATCATGGACGCGGTGGTGTCGCGGGACGGATACTGGCCTCCCGTGC-GCCCCG
AtITS2           --ATCGTCGTCCCTCACCATCCTTTGCTGA--------------TGC------GGGACGGAAGCTGGTCTCCCGTGTGTTACCG
                       **  ***   *                           *         *  ****   *** ********       *

TcoITS2          ----TGTG-GTTGGCCAAAATAGGAGTC-CCTTCG--ATGGACGCACGAACTAGTGGTGGTCGTAAA--AACCCTCGTTCTTTG
TciITS2          ----TGTG-GTTGGCCAAAACAGGAGTA-CCTTCG--ATGGACGCACGAACTAGTGGTGGTCGTAAA--AACCCTCGTTCTTTG
AaITS2           ----CGTG-GTTGGCCGAAATAGGAGTC-CCTTCG--ATGGACGCACGAACTAGTGGTGGTCGTAAA--AACCCTCGTCTTTTG
CsITS2           ----CGTG-GTTGGCTAAAATAGGAGTC-CCTTTG--ATGGATGCATAAACTAGTGGTGGTCGGAAA--AACCCTCGTCTTTTG
HaITS2           ----CGTG-GTTGGCCTAAATAGGAGTC-TCCTCGCGAGGGACGCACGG-CTAGTGGTGGTTGATAA--GACAGTCGTCTCGTG
NtITS2           AGCATGCG-GTTGGCCTAAATGCGAGT--CCACGGCGACGGACGTCACGACAAGTGGTGGTTGAAACTCAACTCTCGTAATGTC
AtITS2           ----CACGCGTTGGCCTAAATCCGAGCCAAGGACGCCTGGAGCGTACCGACATGCGGTGGT-GAACTTGATCCATTACATTTTA
                        * ******  ***   ***        *    *   *      *  * ****** *        *  *       *

TcoITS2          TTCTGTGT---TAGTCGTAAGGAAAAACTCT-TCAAATAACCC-AATGTGTTGTCTTAGGATGACGCTTCGACC----------
TciITS2          TTCTGTGC---TAGTCGCAAGGAAAAACTCT-TCAAATAACCCCAATGTGTTGTCTTAGGATGACGCTTCGACC----------
AaITS2           TTTCGTGCCGTTAGTCGCAAGGGAAA-CTCT-AAGAA-AACCCCAACGTGTCGTCTTTTGACGGCGCTTCGA------------
CsITS2           TTTCGTGTTGATAGTCGCAAGGGAAA-CTCT-TTAAA-AACCCCAATGTGTC----TTTGACGACGCTTCGA------------
HaITS2           TCGTGCGTTTACTTTCTTGAGAGTAGATGCTCTTAAAGTACCCTGATGTGTTGTCTTATGACGATGCTTCGATCGCGACCCCAG
NtITS2           GCGGCTCCGACCCGTCGCACGTTTGGGCTCC----ACGACCCTTGTTGCGCT----TA----GACGCTCCGACCGCGACGCCAG
AtITS2           TCGGTCGCTCTTGTCCGGAAGCTGTAGATGACCCAAA--GTCCATATA------------------------------------
                                *    *              *     *

TcoITS2          --------------------
TciITS2          --------------------
AaITS2           --------------------
CsITS2           --------------------
HaITS2           GTCAGGCGGGACTA------
NtITS2           TCAGCGGGACTACCCTGAGT
AtITS2           --------------------
```

Supplemental Figure S1.

```
Tci_rbcL ATGTCACCACAAACAGAGACTAAAGCAAGTGTTGGATTCAAAGCTGGGGTTAAAGATTATAAATTGACTTATTATACTCCTGAGTATGAAACCAAGGATA
Tco_rbcL ATGTCACCACAAACAGAGACTAAAGCAAGTGTTGGATTCAAAGCTGGGGTTAAAGATTATAAATTGACTTATTATACTCCTGAGTATGAAACCAAGGATA
Aa_rbcL  ATGTCACCACAAACAGAGACTAAAGCAAGTGTTGGATTCAAAGCTGGGGTTAAAGATTATAAATTGACTTATTATACTCCTGAGTATGAAACCAAGGATA
Ha_rbcL  ATGTCACCACAAACAGAGACTAAAGCAAGTGTTGGATTCAAAGCTGGTGTTAAAGATTATAAATTGACTTATTATACTCCTGAATATGAAACCAAGGATA
Nt_rbcL  ATGTCACCACAAACAGAGACTAAAGCAAGTGTTGGATTCAAAGCTGGTGTTAAAGAGTACAAATTGACTTATTATACTCCTGAGTACCAAACCAAGGATA
Cs_rbcL  ATGTCACCACAAACAGAGACTAAAGCAAGTGTTGGATTCAAAGCTGGTGTAAAAGACTACAAATTGACTTATTATACTCCTGAGTACCAAACCAAAGATA
At_rbcL  ATGTCACCACAAACAGAGACTAAAGCAAGTGTTGGGTTCAAAGCTGGTGTTAAAGAGTATAAATTGACTTACTATACTCCTGAATATGAAACCAAGGATA
         ********************************** *********** ** ***** ** *********** *********** **  ******* ****

Tci_rbcL CTGATATCTTGGCAGCATTTCGAGTAACTCCTCAACCTGGAGTTCCGCCTGAAGAAGCAGGGGCCGCAGTAGCTGCCGAATCTTCTACTGGTACATGGAC
Tco_rbcL CTGATATCTTGGCAGCATTTCGAGTAACTCCTCAACCTGGAGTTCCGCCTGAAGAAGCAGGGGCCGCAGTAGCTGCCGAATCTTCTACTGGTACATGGAC
Aa_rbcL  CTGATATCTTGGCAGCATTTCGAGTAACTCCTCAACCTGGAGTTCCGCCTGAAGAAGCAGGGGCCGCAGTAGCTGCCGAATCTTCTACTGGTACATGGAC
Ha_rbcL  CTGATATCTTGGCAGCATTTCGAGTAACTCCTCAACCTGGAGTTCCGCCTGAAGAAGCAGGGGCCGCAGTAGCTGCCGAATCTTCTACTGGTACATGGAC
Nt_rbcL  CTGATATATTGGCAGCATTCCGAGTAACTCCTCAACCTGGAGTTCCACCTGAAGAAGCAGGGGCCGCGGTAGCTGCCGAATCTTCTACTGGTACATGGAC
Cs_rbcL  CTGATATCTTGGCAGCATTCCGAGTAACTCCTCAACCCGGAGTTCCACCTGAAGAAGCAGGGGCTGCGGTAGCTGCGGAATCTTCTACTGGTACATGGAC
At_rbcL  CTGATATCTTGGCAGCATTCCGAGTAACTCCTCAACCTGGAGTTCCACCTGAAGAAGCAGGGGCTGCGGTAGCTGCTGAATCTTCTACTGGTACATGGAC
         ******* *********** ***************** ******** ***************** ** ******** **********************

Tci_rbcL AACTGTGTGGACCGATGGACTTACGAGCCTTGATCGTTACAAAGGGCGCTGCTATGGAATTGAGCCTGTTCCTGGAGAAGAGAATCAATATATTGCTTAT
Tco_rbcL AACTGTGTGGACCGATGGACTTACGAGCCTTGATCGTTACAAAGGGCGCTGCTATGGAATTGAGCCTGTTCTTGGAGAAGAGAATCAATATATTGCTTAT
Aa_rbcL  AACTGTGTGGACCGATGGACTTACGAGCCTTGATCGTTACAAAGGGCGATGCTATGGAATTGAGCCTGTTCCTGGAGAAGAGAATCAATATATTTGCTAT
Ha_rbcL  AACTGTATGGACCGATGGACTTACCAGCCTTGACCGTTACAAAGGCCGATGCTATGGACTTGAGCCTGTTCCTGGAGAAGACAATCAATTTATTGCTTAT
Nt_rbcL  AACTGTATGGACCGATGGACTTACCAGCCTTGATCGTTACAAAGGGCGATGCTACCGCATCGAGCGTGTTGTTGGAGAAAAAGATCAATATATTGCTTAT
Cs_rbcL  AACTGTGTGGACCGATGGACTTACCAGCCTTGATCGCTACAAGGGGCGATGCTACCGCATCGAGCGCGTTATTGGAGAAAAAGATCAATATATTGCTTAT
At_rbcL  AACTGTGTGGACCGATGGGCTTACCAGCCTTGATCGTTACAAAGGACGATGCTACCACATCGAGCCCGTTCCAGGAGAAGAAACTCAATTTATTGCGTAT
         ****** *********** ***** ******** ** ***** ** ** *****      * ****  ***   ****** *   ***** ****   ***

Tci_rbcL GTAGCTTACCCATTAGACCTTTTTGAAGAAGGTTCTGTTACTAACATGTTTACTTCCATTGTAGGTAACGTATTTGGTTTCAAAGCCCTGCGTGCTCTAC
Tco_rbcL GTAGCTTACCCATTAGACCTTTTTGAAGAAGGTTCTGTTACTAACATGTTTACTTCCATTGTAGGTAACGTATTTGGTTTCAAAGCCCTGCGTGCTCTAC
Aa_rbcL  GTAGCTTACCCATTAGACCTTTTTGAAGAAGGTTCTGTTACTAACATGTTTACTTCCATTGTAGGTAACGTATTTGGTTTCAAAGCCCTGCGTGCTCTAC
Ha_rbcL  GTAGCGTACCCATTAGACCTTTTTGAAGAAGGTTCTGTTACTAACATGTTTACTTCCATTGTAGGTAATGTATTTGGGTTCAAAGCCCTGCGTGCTCTAC
Nt_rbcL  GTAGCTTACCCTTTAGACCTTTTTGAAGAAGGTTCTGTTACCAACATGTTTACTTCCATTGTAGGTAACGTATTTGGGTTCAAAGCCCTGCGCGCTCTAC
Cs_rbcL  GTAGCTTACCCTTTAGACCTTTTTGAAGAAGGTTCTGTTACCAACATGTTTACTTCCATTGTGGGTAATGTATTTGGGTTCAAAGCACTGCGCGCTCTAC
At_rbcL  GTAGCTTATCCCTTAGACCTTTTTGAAGAAGGTTCGGTTACTAACATGTTTACCTCGATTGTGGGTAATGTATTTGGGTTCAAAGCCCTGGCTGCTCTAC
         ***** ** ** ********************** ***** *********** ** ***** ***** ******** ******** ***   *******

Tci_rbcL GTCTGGAAGATTTGCGAATTCCTACTGCGTATGTTAAAACTTTCCAAGGTCCGCCTCACGGTATCCAAGTTGAAAGAGATAAATTGAACAAGTATGGTCG
Tco_rbcL GTCTGGAAGATTTGCGAATTCCTACTGCGTATGTTAAAACTTTCCAAGGTCCGCCTCACGGTATCCAAGTTGAAAGAGATAAATTGAACAAGTATGGTCG
Aa_rbcL  GTCTGGAAGATTTGCGAATTCCTACTGCGTATGTTAAAACTTTCCAAGGTCCGCCTCACGGTATCCAAGTTGAAAGAGATAAATTGAACAAGTATGGTCG
Ha_rbcL  GTCTGGAAGATTTGCGAATCCCGACTGCGTATGTTAAAACTTTCGACGGTCCGCCTCACGGTATCCAAGTTGAAAGAGATAAATTGAACAAGTATGGTCG
Nt_rbcL  GTCTGGAAGATCTGCGAATCCCTCCTGCTTATGTTAAAACTTTCCAAGGTCCGCCTCATGGGATCCAAGTTGAAAGAGATAAATTGAACAAGTATGGTCG
Cs_rbcL  GTCTGGAAGATTTACGAATCCCTACGGCTTATATTAAAACTTTTCAAGGCCCGCCTCACGGAATCCAAGTTGAGAGAGATAAATTGAACAAGTATGGTCG
At_rbcL  GTCTAGAGGATCTGCGAATCCCTCCTGCTTATACTAAAACTTTCCAAGGACCACCTCATGGTATCCAAGTTGAAAGAGATAAATTGAACAAGTATGGACG
         **** ** *** * ***** **   * ** ***  *********   * ** ** ***** ** *********** ********************** **

Tci_rbcL TCCTCTGTTGGGATGTACTATTAAACCTAAATTGGGGTTATCCGCTAAAAACTACGGTAGAGCTGTTTATGAATGTCTTCGTGGTGGCCTTGATTTTACT
Tco_rbcL TCCTCTGTTGGGATGTACTATTAAACCTAAATTGGGGTTATCCGCTAAAAACTACGGTAGAGCTGTTTATGAATGTCTTCGTGGTGGCCTTGATTTTACT
Aa_rbcL  TCCTCTGTTGGGATGTACTATTAAACCTAAATTGGGGTTATCTGCTAAAAACTACGGTAGAGCTGTTTATGAATGTCTTCGTGGTGGCCTTGATTTTACT
Ha_rbcL  TCCCCTGTTGGGATGTACTATTAAACCGAAATTGGGGTTATCCGCTAAAAACTACGGTAGAGCTTGTTATGAATGTCTTCGTGGTGGCCTTGATTTTACT
Nt_rbcL  TCCCCTGTTGGGATGTACTATTAAACCTAAATTGGGGTTATCTGCTAAAAACTACGGTAGAGCTGTTTATGAATGTCTTCGCGGTGGACTTGATTTTACC
Cs_rbcL  TCCTCTGTTGGGATGTACTATTAAACCTAAATTGGGGTTATCTGCTAAAAACTACGGTAGAGCGGTTTATGAATGTCTTCGCGGTGGACTTGATTTTACC
At_rbcL  TCCCCTATTAGGATGTACTATTAAACCAAAATTGGGGTTATCCGCGAAAAACTATGGTAGAGCAGTTTATGAATGTCTACGTGGTGGACTTGATTTTACC
         *** ** ** ***************** ************** ** ******** ********    ************ ** ***** ***********

Tci_rbcL AAAGATGATGAGAACGTGAACTCCCAACCATTTATGCGTTGGAGAGACCGTTTCTTATTTTGTGCCGAAGCTATTTATAAATCACAAGCTGAAACAGGTG
Tco_rbcL AAAGATGATGAGAACGTGAACTCCCAACCATTTATGCGTTGGAGAGACCGTTTCTTATTTTGTGCCGAAGCTATTTATAAATCACAAGCTGAAACAGGTG
Aa_rbcL  AAAGATGATGAGAACGTGAACTCCCAACCATTTATGCGTTGGAGAGACCGTTTCTTATTTTGTGCCGAAGCTATTTTTAAATCACAAGCTGAAACAGGTG
Ha_rbcL  AAAGATGATGAGAACGTGAACTCCCAACCATTTATGCGTTGGAGAGACCGTTTCTTATTTTGTGCCGAAGCTCTTTATAAAGCACAAGCTGAAACAGGTG
Nt_rbcL  AAAGATGATGAGAACGTGAACTCACAACCATTTATGCGTTGGAGAGATCGTTTCTTATTTTGTGCCGAAGCACTTTATAAAGCACAGGCTGAAACAGGTG
Cs_rbcL  AAAGATGATGAGAACGTAAACTCACAACCATTTATGCGTTGGAGAGACCGTTTCTTATTTTGTGCCGAAGCACTTTATAAAGCACAGGCTGAAACAGGTG
At_rbcL  AAAGATGATGAGAATGTGAACTCCCAACCATTTATGCGTTGGAGAGACCGTTTCTTATTTTGTGCCGAAGCTATTTATAAATCACAGGCTGAAACAGGTG
         ************** ** ***** ********************** ***********************  *** **** **** *************

Tci_rbcL AAATCAAAGGGCATTACTTGAATGCTACTGCGGGTACATGCGAAGAAATGATGAAAAGGGCTATATTTGCCAGAGAATTGGGAGTTCCTATTGTAATGCA
Tco_rbcL AAATCAAAGGGCATTACTTGAATGCTACTGCGGGTACATGCGAAGAAATGATGAAAAGGGCTATATTTGCCAGAGAATTGGGAGTTCCTATTGTAATGCA
Aa_rbcL  AAATCAAAGGGCATTACTTGAATGCTACTGCGGGTACATGCGAAGACATGATGAAAAGGGCTGTATTTGCCAGAGAATTGGGAGTTCCTATCGTAATGCA
Ha_rbcL  AAATCAAAGGGCATTACTTGAATGCTACTACGGGTAATTGCGAAGATATGATGAAAAGGGCTGTATTTGCTAGAGAATTGGGAGTTCCTATCGTAATGCA
Nt_rbcL  AAATCAAAGGGCATTACTTGAATGCTACTGCAGGTACATGCGAAGAAATGATCAAAAGAGCTGTATTTGCTAGAGAATTGGGCGTTCCGATCGTAATGCA
Cs_rbcL  AAATCAAAGGACATTACTTGAATGCTACTGCAGGTACATGCGAAGAAATGATGAAAAGAGCTATATTTGCTAGAGAATTGGGAGTTCCGATTGTAATGCA
At_rbcL  AAATCAAAGGGCATTATTTGAATGCTACTGCGGGTACATGCGAAGAAATGATCAAAAGAGCTGTATTTGCCAGAGAATTGGGAGTTCCTATCGTAATGCA
         ********** ***** ************ * ****  ******** ***** ***** *** ******* *********** ***** ** ********
```

[continued to the next page]

Supplemental Figure S1

```
[continued from the previous page]

Tci_rbcL TGACTACCTAACAGGGGGATTCACTGCAAATACTAGCTTGGCTCATTATTGCCGAGATAATGGCCTACTTCTTCACATCCACCGCGCAATGCATGCAGTT
Tco_rbcL TGACTACCTAACAGGGGGATTCACTGCAAATACTAGCTTGGCTCATTATTGCCGAGATAATGGCCTACTTCTTCACATCCACCGCGCAATGCATGCAGTT
Aa_rbcL  TGACTACCTAACAGGTGGATTCACTGCAAATACTACCTTGGCTCATTATTGCCGAGATAATGGCTTACTTCTTCACATCCACCGCGCAATGCATGCAGTT
Ha_rbcL  TGACTACCTAACAGGTGGATTCACTGCAAATACTAGCTTGTCTCAGTATTGCCGAGATAATGGTCTACTTCTTCACATCCACCGCGCAATGCATGCGGTT
Nt_rbcL  TGACTACTTAACGGGGGGATTCACCGCAAATACTAGCTTGGCTCATTATTGCCGAGATAATGGTCTACTTCTTCACATCCACCGTGCAATGCATGCGGTT
Cs_rbcL  TGACTACTTAACAGGGGGATTCACTGCAAATACTTCTTTGGCTCATTATTGCCGAGATAATGGTCTACTTCTTCACATCCACCGTGCAATGCATGCAGTT
At_rbcL  TGACTACTTAACAGGGGGATTCACCGCAAATACTAGTTTGTCTCATTATTGCCGAGATAATGGCCTACTTCTTCACATCCACCGTGCAATGCACGCTGTT
         ******* **** ** ******** *********   *** **** *****************  ******************  ******** ** ***

Tci_rbcL ATTGATAGACAGAAGAACCATGGTATACACTTCCGTGTACTAGCTAAAGCGTTACGTATGTCTGGTGGAGATCATATCCATTCCGGTACCGTAGTAGGTA
Tco_rbcL ATTGATAGACAGAAGAACCATGGTATACACTTCCGTGTACTAGCTAAAGCGTTACGTATGTCTGGTGGAGATCATATCCATGCCGGTACCGTAGTAGGTA
Aa_rbcL  ATTGATAGACAGAAGAATCATGGTATGCACTTCCGTGTACTAGCTAAAGCGTTACGTATGTCTGGTGGAGATCATATCCATGCCGGTACCGTAGTAGGTA
Ha_rbcL  ATTGATAGACAGAAGAATCATGGTATGCACTTCCGTGTACTAGCTAAAGCGTTACGTATGTCCGGTGGAGATCACATTCATTCCGGTACCGTAGTAGGTA
Nt_rbcL  ATTGATAGACAGAAGAATCATGGTATCCACTTCCGGGTATTAGCAAAAGCGTTACGTATGTCTGGTGGAGATCATATTCACTCTGGTACCGTAGTAGGTA
Cs_rbcL  ATTGATAGACAGAAGAATCATGGTATGCACTTCCGTGTACTAGCTAAAGCGTTACGTCTGTCTGGTGGAGATCATATTCACGCGGGTACCGTAGTAGGTA
At_rbcL  ATTGATAGACAGAAGAATCATGGTATGCACTTCCGTGTACTAGCTAAAGCTTTACGTCTATCTGGTGGAGATCATATTCACGCGGGTACAGTAGTAGGTA
         ***************** ******** ******** *** **** ***** ****** * ** *********** ** **  * ***** **********

Tci_rbcL AACTTGAAGGGGAAAGAGAGATCACTTTGGGCTTTGTTGATTTACTACGTGATGATTTTATTGAAAAAGATAGAAGTCGCGGTATTTATTTCACCCAAGA
Tco_rbcL AACTTGAAGGGGAAAGAGAGATCACTTTGGGCTTTGTTGATTTACTACGTGATGATTTTATTGAAAAAGATAGAAGTCGCGGTATTTATTTCACCCAAGA
Aa_rbcL  AACTTGAAGGGGAAAGAGAGATCACTTTGGGCTTTGTTGATTTATTACGTGATGATTTTATTGAAAAAGATAGAAGTCGCGGTATTTATTTCACCCAAGA
Ha_rbcL  AACTTGAAGGGGAAAGAGAAATCACTTTGGGCTTTGTTGATTTACTGCGTGATGATTTTATTGAAAAAGATAGAAGTCGCGGTATTTATTTCACCCAAGA
Nt_rbcL  AACTTGAAGGTGAAAGAGACATAACTTTGGGCTTTGTTGATTTACTGCGTGATGATTTTGTTGAACAAGATCGAAGTCGCGGTATTTATTTCACTCAAGA
Cs_rbcL  AACTTGAAGGGGAAAGAGAGATTACTTTGGGCTTTGTTGACTTACTACGTGATGATTTTGTTGAACAAGACCGAAGTCGCGGTATTTATTTCACTCAAGA
At_rbcL  AACTTGAAGGAGACAGGGAGTCAACTTTGGGCTTTGTTGATTTACTGCGCGATGATTATGTTGAAAAAGATCGAAGCCGCGGTATCTTTTTCACTCAAGA
         ********** ** ** **    ****************  *** * ** ******* * ***** ****  **** ******** * ****** *****

Tci_rbcL TTGGGTGTCTCTACCAGGTGTTCTGCCGGTAGCTTCGGGCGGTATTCACGTTTGGCATATGCCTGCTCTGACCGAGATCTTTGGGGATGATTCCGTACTA
Tco_rbcL TTGGGTGTCTCTACCAGGTGTTCTGCCGGTAGCTTCGGGCGGTATTCACGTTTGGCATATGCCTGCTCTGACCGAGATCTTTGGGGATGATTCCGTACTA
Aa_rbcL  TTGGGTGTCTCTACCGGGTGTTCTGCCGGTAGCTTCGGGCGGTATTCACGTTTGGCATATGCCTGCTCTGACCGAGATCTTTGGAGATGATTCCGTACTA
Ha_rbcL  TTGGGTCTCTCTACCAGGTGTTCTGCCTGTAGCTTCGGGGGGTATTCACGTTTGGCATATGCCTGCTCTAACCGAGATCTTTGGGGATGATTCCGTACTA
Nt_rbcL  TTGGGTCTCTTTACCAGGTGTTCTACCCGTGGCTTCAGGAGGTATTCACGTTTGGCATATGCCTGCTCTGACCGAGATCTTTGGGGATGATTCCGTACTA
Cs_rbcL  TTGGGTCTCTTTACCAGGTGTTCTGCCTGTTGCTTCAGGGGGTATTCACGTTTGGCATATGCCTGCTCTGACCGAGATCTTTGGGGATGATTCCGTACTA
At_rbcL  TTGGGTCTCACTACCTGGTGTTCTGCCTGTGGCTTCAGGGGGTATTCACGTTTGGCATATGCCTGCTTTGACCGAGATCTTTGGAGATGATTCTGTACTA
         ****** **  **** ******** ** ** ***** ** *************************** * ************** ******** ******

Tci_rbcL CAGTTCGGTGGCGGAACTTTAGGCCACCCTTGGGGAAATGCACCTGGTGCCGTAGCTAACCGAGTAGCTCTAGAAGCATGTGTACAAGCTCGTAATGAGG
Tco_rbcL CAGTTCGGTGGCGGAACTTTAGGCCACCCTTGGGGAAATGCACCTGGTGCCGTAGCTAACCGAGTAGCTCTAGAAGCATGTGTACAAGCTCGTAATGAGG
Aa_rbcL  CAGTTCGGTGGCGGAACTTTAGGCCACCCTTGGGGAAATGCACCTGGTGCCGTAGCTAACCGAGTAGCTCTAGAAGCATGTGTACAAGCTCGTAATGAAG
Ha_rbcL  CAGTTCGGTGGAGGAACTTTAGGGCACCCTTGGGAAAATGCACCTGTCGTCGTAGCTAACCGAGTAGCTCTAGAAGCATGTGTACAAGCTCGTAATGAGG
Nt_rbcL  CAGTTCGGTGGAGGAACTTTAGGACATCCTTGGGGTAATGCGCCAGGTGCCGTAGCTAATCGAGTAGCTCTAGAAGCATGTGTAAAAGCTCGTAATGAAG
Cs_rbcL  CAGTTCGGGGGAGGAACTTTAGGGCACCCTTGGGGTAATGCGCCAGGTGCCGTAGCTAATCGAGTCGCTCTAGAAGCATGTGTACAAGCTCGTAACGAAG
At_rbcL  CAATTCGGTGGAGGAACTTTAGGCCACCCTTGGGGAAATGCACCGGGTGCCGTAGCCAACCGAGTAGCTCTGGAAGCATGTGTACAAGCTCGTAATGAGG
         ** ***** ** *********** ** *******  ***** ** *  * ****** ** ***** ***** ************ ********** ** *

Tci_rbcL GACGCGATCTTGCTACCGAGGGTAATGAAATTATCCGCGAAGCTACCAAATGGAGTCCTGAACTAGCTGCTGCTTGTGAAGTATGGAAGGAGATCAAATT
Tco_rbcL GACGCGATCTTGCTACCGAGGGTAATGAAATTATCCGCGAAGCTACCAAATGGAGTCCTGAACTAGCTGCTGCTTGTGAAGTATGGAAGGAGATCAAATT
Aa_rbcL  GACGCGATCTTGCTACCGAGGGTAATGAAATTATCCGCGAAGCTACCAAATGGAGTCCTGAACTAGCTGCTGCTTGTGAAGTATGGAAGGAGATCAAATT
Ha_rbcL  GACGCGATCTTGCTACTGAGGGTAATGAAATTATCCGTGAGCATAGCAAATGGAGTCCTGAACTAGCTGCTGCTTGTGAAGTATGGAAGGAGATCAAATT
Nt_rbcL  GACGTGATCTTGCTCAGGAAGGTAATGAAATTATTCGCGAGGCTTGCAAATGGAGCCCGGAACTAGCTGCTGCTTGTGAAGTATGGAAAGAGATCGTATT
Cs_rbcL  GACGTGATCTTGCTCGGGAAGGTAATGAAATTATTCGCGAGGCTTGCAAATGGAGCCCTGAACTATCTGCTGCTTGTGAGGTATGGAAGGAGATCCGATT
At_rbcL  GACGTGATCTTGCAGTCGAGGGTAATGAAATTATCCGTGAAGCTTGCAAATGGAGTCCTGAACTAGCTGCTGCTTGTGAAGTATGGAAAGAGATCACATT
         **** ********    ** ************** ** **   *  ********* ** ****** ************* ******** ******  ***

Tci_rbcL TGAATTCCAGGCAATGGATACTTTGGATGGGGATAAAGATAAGGATAAAAAGAGATAA
Tco_rbcL TGAATTCCAGGCAATGGATACTTTGGATGGGGATAAAGATAAGGATAAAAAGAGATAA
Aa_rbcL  TGAATTCCAGGCAATGGATACTTTGGATGGGGATAAAGATAAGGATAAAAAGAGATAA
Ha_rbcL  TGAGTTCCAGGCAATGGATCCTTTGGATACGGATAAGGATAAAGATAAGAAGAGATAA
Nt_rbcL  TAATTTTGCAGCAGTGGACGTTTTGGAT--AAGTAA----------------------
Cs_rbcL  TGAATTTAAACCAGTGGATACCTTGGATCCAGGTACAGCTTAA---------------
At_rbcL  TAACTTCCCAACCATCGATAAATTAGAT--GGCCAAGAGTAG----------------
         * * **     *  * **    ** ***      *
```

Supplemental Figure S1.

```
Tco_matK ATGGAGAAATTCAAAAGCTATTTAGGGCTAGATAGATCTCAACAACACTCTTTCTTATATCCACTTA---TCTTTCAGGAGTATATTTATGTACTTGCTC
Tci_matK ATGGAGAAATTCAAAAGCTATTTAGGGCTAGATAGATCTCAACAACACTCTTTCTTATATCCACTTA---TCTTTCAGGAGTATATTTATGTACTTGCTC
Aa_matK  ATGGAGAAATTCCAAAGCTATTTAGGGCTAGATAGATCTCAACAACACTCTTTCTTATATCCACTTA---TCTTTCAGGAGTATATTTATGTACTTGCTC
Ha_matK  ATGGAGAAATTCCAAAG---------GCTAGATAGATCTCA------CTACTTCTTATATCCACTTA---TCTTTCAGGAGTATATTTATGTACTTGCTC
Nt_matK  ATGGAAGAAATCCAAAGATATTTACAGCCAGATAGATCGCAACAACACAACTTCCTATATCCACTTA---TCTTTCAGGAGTATATTTATGCACTTGCTC
Cs_matK  ------------------------------ATGGGGGAGTCTAAGGAGAGTCTAGCGAATCCGTTTAAAGTTTCCCAGGAGTCTATTTATGCACTTGCTC
At_matK  ATGGATAAATTTCAAGGATATTTAGAGTTCGATGGGGCTCGGCAACAGAGTTTTCTATATCCACTTT---TTTTTCGGGAGTATATTTATGTACTTGCTT
                                           *                 *     ****  **    * *  * ***** ******** *******

Tco_matK ATGATCATGGTTTAAA---------TAGA------TCTATTTTGTTGGAAAATGTAGGTTATGACAATAAATTCAGCTTACTAATTGTGAAACGTTTAAT
Tci_matK ATGATCATGGTTTAAA---------TAGA------TCTATTTTGTTGGAAAATGTAGGTTATGACAATAAATTCAGCTTACTAATTGTGAAACGTTTAAT
Aa_matK  ATGATCATGGTTTAAA---------TAGA------TCTATTTTGTTGGAAAATGTAGGTTATGACAATAAATTCAGCTTACTAATTGTGAAACGTTTAAT
Ha_matK  ATGATCATGGTTTAAA---------TGGA------TCGATTTTGTTGGAAAATGCAGGTTATGACAATAAATCCAGTTTACTAATTGTGAAACGTTTAAT
Nt_matK  ATGATCATGGTTTAAATAGA---AATAGG------TCGATTTTGTTGGAAAATCCAGGTTATAACAATAAATTAAGTTTCCTAATTGTGAAACGTTTAAT
Cs_matK  ATGATCGTGGTTTAAA---------TAGG------TCGATTTCTTTTAAAAATACCGGGTATGAAAAAAAATTGAGTTTTCAAATTGTGAAACGCTTAAT
At_matK  ATGATCATGGTTTAAATAGATTAAATAGAAATCGCTATATTTTCTTGGAAAATGCGGATTATGACAAAAAATATAGTTCACTAATTACGAAACGCTTAAT
         ****** *********         * *       *  ****  **  *****   *  *** * ** ****  ** *  * ****  ****** *****

Tco_matK CATTCGAATGTATCAACAGAATCATTTTATTCTTTCTGTTAATGATTCTAAACAA---------TTGGGGCACAACAATATTTTTTATTCGCAAGTAATG
Tci_matK CATTCGAATGTATCAACAGAATCATTTTATTCTTTCTGTTAATGATTCTAAACAA---------TTGGGGCACAACAATATTTTTTATTCGCAAGTAATG
Aa_matK  CATTCGAATGTATCAACAGAATCATTTTATTCTTTCTGTTAATGATTCTAAACAA---------TTGGGGCACAACAATAATTTTTATTCGCAAGTAATG
Ha_matK  CATTCGAATGTATCAACAGAATCATTTGATTCTTTCTGTTAATGATTCTAAACAGACTGCATTTTGGGGCACAACAAGAATTTTTATTCGCAAGTAATG
Nt_matK  TACTCGAATGTATCAACAGAATCATTTTCTTATTTCTACTAATGATTCTAACAAAAATTCATTTTGGGGTGCAACAAGAGTTTGTATTCTCAAATGATA
Cs_matK  TACTCGAATTTATCAACAGATCTATTTTAGTACTTATTCT------TCTAACAAAAGTCAAATTTTGGGCCCAACAAGAGTTTGTATTCTAAACTGCTA
At_matK  TTTGCGAATGTATGAACAGAATCGTTTGATTATTCCCACTAAGGATGTGAACCAAAATTCCTTTTTGGGGCATACCAGTCTTTTCTATTATCAAATGATA
             ***** *** ******    ***   *  *     *         **  *          ** ***   * **    *** ****   ** *  *

Tco_matK TCAGAGGTTTCTTCAATCATTCTGGAAATTCCATTGTCTCTGCGATTAATATCTTCCC---------TAGAAAAGAAA------GGGGTAGTTAAATTCG
Tci_matK TCAGAGGTTTCTTCAATCATTCTGGAAATTCCATTGTCTCTGCGATTAATATCTTCCC---------TAGAAAAGAAA------GGGGTAGTTAAATTCG
Aa_matK  TCAGAGGTTTCTTCAATTATTCTGGAAATTCCATTGTCTCTGCGATTAATATCTTCCC---------TAGAAAAGAAAAAGAAAGGGTTAGTTAAATTCG
Ha_matK  TCAGAGGTTTCTTCAACCATTATGGAAATTCCATTGTCTCTGCGATTAATATCTTCCC---------TAGAAAGGAAA------GGGGTAGTTAAATCCG
Nt_matK  TCAGAGGGATTTGCGTTTATTGTGGAAATTCCGTTTTCTCTACGATTAATATCTTCTTTATCTTCTTTCGAAGGCAAA------AAGATTTTTAAATCTT
Cs_matK  TCAGAGGGGTTTGCTTTTATTGTGGAAATTCCGTTTTCTTTACGATTCATAT---------------TAGAGCGCAAA------AAAATATTAAAATCTC
At_matK  TCTGTTTTATTTGCAGTGATTGTCGAAATTCCATTTTCCCTAAGATTAGGATCCTCTT---------TTCAAGGAAAA------CAATTAAAAAAATCTT
         ** *     * * *    *** * ******** ** **  *  ****   **               *  *    ***          *    ****

Tco_matK ATAATTTACGATCAATTCATTCAATATTTTCTTTTTTAGAGGACAATTTTTCACATTTAAATTATGTATTAGATATACTAATACCTTACCCAGCCCATCT
Tci_matK ATAATTTACGATCAATTCATTCAATATTTTCTTTTTTAGAGGACAATTTTTCACATTTAAATTATGTATTAGATATACTAATACCTTACCCAGCCCATCT
Aa_matK  ATAATTTACGATCAATTCATTCAATATTTTCTTTTTTAGAGGACAACTTTTCACATTTAAATTATGTATTAGATATACTAATACCTTACCCAGCCCATCT
Ha_matK  ATAATTTACGATCAATTCATTCCATATTTTCTTTTTTAGAGGACAACTTTTCACATTTAAATTATGTATTAGATATACTAATACCTTACCCAGCTCATCT
Nt_matK  ATAATTTACGATCAATTCATTCAACATTTCCTTTTTTAGAGGACAATTTTTCACATCTAAATTATGTATTAGATATACTAATACCCTACCCTGTTCATCT
Cs_matK  AGAATTTACGATCAATTCATTCAATATTTCCTTTTTTAGAGGACAATTTCTCACATTTAAATTATGTATTAGATATACTAATACCCTACCCCCCTCATCT
At_matK  ATAATTTACAATCAATTCATTCAATATTTCCCTTTTTAGAAGACAAATTAGGACATTTTAATTATGTGTTAGATGTACTAATACCTTACCCCATCCATCT
         * ******* ************ * **** * ******** ***** **   **** * ******** ****** ********** *****    *****

Tco_matK GGAAATCTTGGTTCAGGCTCTTCGCTATTGGATAAAAGATGCTTCCTCTTTGCATTTATTAAGATTCTTTCTCCATGAGTGTCATAATTGGGATAGTCTT
Tci_matK GGAAATCTTGGTTCAGGCTCTTCGCTATTGGATAAAAGATGCTTCCTCTTTGCATTTATTAAGATTCTTTCTCCATGAGTGTCATAATTGGGATAGTCTT
Aa_matK  GGAAATCTTGGTTCAGGCTCTTCGCTATTGGATAAAAGATGCTTCCTCTTTGCATTTATTAAGATTCTTTCTCCATGAGTGTCATAATTGGGATAGTCTT
Ha_matK  GGAAATCTTGGTTCAGGCTCTTCGCTATTGGATCAAAGATGCTTCCTCTTTGCATTTATTAAGATTCTTTCTCCATGAGTGTCATAATTGGGATAGTCTT
Nt_matK  GGAAATCTTGGTTCAAACTCTTCGCTATTGGGTAAAAGATGCCTCTTCTTTACATTTATTACGATTCTTTCTCCATGAATTTTGGAATTTGAATAGTCTT
Cs_matK  GGAAATCTTGGTTCAAACTCTTCACTTTTGGGTGAAAGACGCTTCTTCTTTGCATCTATTACGATTCTTTCTCCACGAGTATTGCAATTTTAATAGTTTC
At_matK  AGAAATCTTGGTTCAAACCCTACGTTACCGGGTAAAAGATGCCTCTTCTTTGCATTTTTTTCGGTTCTGTTTATACGAGTATTGTAATTGGAAGAATTT-
          **************  * ** *  *   ** * ***** ** ** ***** *** * **  * **** * *  * ** * *   ****   * * * *

Tco_matK ATTACTTCAAATTCAAAGAAAGTTAGTTCTTCTTTTTCAAAAAGAAAAAACAGATTATTCTTCTTCCTATATACTTTTCATGTAGGTGAATATGAATCTG
Tci_matK ATTACTTCAAATTCAAAGAAAGTTAGTTCTTCTTTTTCAAAAAGAAAAAACAGATTATTCTTCTTCCTATATACTTTTCATGTAGGTGAATATGAATCTG
Aa_matK  ATTACTTCAAATTCAAAGAAAGTTAGTTCTTCTTTTTCAAAAAGAAAAAACAGATTATTCTTCTTCCTATATACTTTTCATGTATGTGAATATGAATCTG
Ha_matK  ATTACTTCAAATTCAAAGAAAGCCAGTTCTTTTTTTTCAAAAAGAAATCACAGACTATTCTTCTTCCTATATACTTCTTATGTATGTGAATATGAATCTG
Nt_matK  ATTACTTCAA------AGAAGCCCGGTTACTCCTTTTCAAAAAAAAATCAAAGATTCTTCTTCTTCTTATATAATTCTTATGTATATGAATGCGAATCCA
Cs_matK  ATTACTCCAA------AGAGGTCCCGTTCCCCTTTTTCAAAGATAAATCAAAGATTTTTCTTCTTCTTATATAACTCTTATGTATGTGAATACGAATCCA
At_matK  -TTATATTAAA-----AAAAAATCAATTTT--------------GAATCCAAGATTTTTCTTGTTCTTATATAATTCTCATGTATGTGAATACGAATCCA
          ***    **      * *       **                 **    *** * ***** *** ******  * * *****  *****  *****

Tco_matK GCTTCCTCTTTCTCCGTAACCAGTCTTCTCACTTACGATCAACATCTTCTGGAGCCCTTATTGAACGAATCAATTTCTATGGAAAAATAGAGCATCTTGC
Tci_matK GCTTCCTCTTTCTCCGTAACCAGTCTTCTCACTTACGATCAACATCTTCTGGAGCCCTTATTGAACGAATCAATTTCTATGGAAAAATAGAGCATCTTGC
Aa_matK  GCTTCCTCTTTCTCCGTAACCAGTCTTCTCACTTACGATCAACATCTTCTGGAGCCCTTATTGAACGAATAAATTTCTATGGAAAAATAGAGCATCTTGC
Ha_matK  GCTTCCTATTTCTCCGTAACCAATCTTCTCACTTACGATCAACATCTTCTGGAGCCCTTATTGAACGAATATATTTCTATGGAAAAATAGAGCATCTTGC
Nt_matK  CTTTCGTCTTTCTACGGAACCAATCTTCTCATTTACGATCAACATCTTTTGGAGCCCTTCTTGAACGAATATATTTCTATGGAAAAATAGAACGTCTTGT
Cs_matK  TTTTTTTATTTCTCCGTAACCAATCTCTTCATTTACGATCAACCAGTTTTGGAGCCCTTTTTGAACGAAACATTTTCTATGGAAAAATAGAATGCTTTGT
At_matK  TCTTTTTTTTTCTACGCAAGCGGTCTTCGCATTTACGATCGACATCTTATGAAGTCCTTTTTGAGCGAATTGTATTCTATGGAAAAATACACCATTTTTT
           **  * ***** ** ** *  ***   ** ******** **   ** ** ** **** **** ****     **************  *     **
```

[continued to the next page]

Supplemental Figure S1.

[continued from the previous page]

```
Tco_matK AGAAGTCTTTGTCAGGTCTTTTCAAGCAAATTTATGGTTGTTCAAAGAT------CCTTTCATGCATTATGTTAGATATCAAGGAAAATCAATTCTTGCT
Tci_matK AGAAGTCTTTGTCAGGTCTTTTCAAGCAAATTTATGGTTGTTCAAAGAT------CCTTTCATGCATTATGTTAGATATCAAGGAAAATCAATTCTTGCT
Aa_matK  AGAAGTCTTTGTCAGGTCTTTTCAAGCAAATTTATGGTTGTTCAAAGAT------CCTTTCATGCATTATGTTAGGTATCAAGGAAAATCCATTCTTGCT
Ha_matK  AGAAGTCTTTGCCAGGACTTTTCAAGCGAATTTATGGTTCTTCAAAGAT------TCTTTCATGCATTATGTTAGGTATCAAGGAAAATCAATTCTTGCT
Nt_matK  AGAAGTCTTTGCTAAGGATTTTCAGGTTACCCTATGGTTATTCAAGGAT------CCTTTCATGCATTATGTTAGGTATCAAGGAAAATCCATTCTGGCT
Cs_matK  AAAAGTCTTTGCTAAGGATTTTAAGGCAAACCTATGTTTGCTCAAGGATGCGGATCTTTCCATGCATTATTTTAGGTATCGGGGAAAATCAATTCTGGCT
At_matK  CAAAGTTTTGTTAATAATTTTCCGGCAATCCTAGGGTTGCTCAAGGAT------CCTTTCATACATTATGTTAGATATCACGGAAGATGCATTCTGGCA
           **** ****  *    ****   *  *   ** * **  **** ***        ** *** ****** **** ****  **** **  ***** **

Tco_matK TCAAAAGGGACGTTTCTTTTGATGAATAAATGGAAATATTACTTTGTCAATTTCTGGAAATCTTATTTTTACCTGTGGCCTCAACCAGGAAGGATTTATA
Tci_matK TCAAAAGGGACGTTTCTTTTGATGAATAAATGGAAATATTACTTTGTCAATTTCTGGAAATCTTATTTTTACCTGTGGCCTCAACCAGGAAGGATTTATA
Aa_matK  TCAAAAGGGACGTTTCTTTTGATGAATAAATGGAAATATTACTTTGTCAATTTCTGGAAATATTATTTTTACCTGTGGCCTCAACCAGGAAGGATTTATA
Ha_matK  TCAAAAGGGACGTTTCTTTTGATGAATAAAAGGAAAGATTACTTTTTCAATTTCTGGAAATCTTATTTTTACCTGTGGTCTTATCCAGGAAGGATTTCTA
Nt_matK  TCAAAAGGGACGTTTCTTTTGATGAATAAATGGAAATTTTACCTTGTCAATTTTTGGCAATGTCATTGTTCTCTGTGCTTTCACACAGGAAGGATCCATA
Cs_matK  TCTAAAGGAACGCTTCTTTTGATGTCTAAATGGAACTATTATTTTGTCAATTTTTGGCAATGTTCTTTTTGTTTGTGGTTCCATACTGAAAGAATCTATA
At_matK  ACAAAGGATACGCCGCTTCTGATGAATAAATGGAAATATTATTTTGTTAATTTATGGCAATGTTATTTTTCGGTATGGTTTCAATCGCAAAAGGTCAATA
          * ** *  ***   *** *****  **** ****   ***  ** * ***** *** *** *  ** **   * **     *  *   **   *   **

Tco_matK TAAACAAATTATCCAATCATTCCCTTGACTTTCTGGGTTATCGTTCAAGTGTGCGGCTAAATCCTTCAATGGTACGCAGTCAAATGCTAGAAAATGCATT
Tci_matK TAAACAAATTATCCAATCATTCCCTTGACTTTCTGGGTTATCGTTCAAGTGTGCGGCTAAATCCTTCAATGGTACGCAGTCAAATGCTAGAAAATGCATT
Aa_matK  TAAACCAATTATCCAATCATTCCCTTGACTTTCTGGGTTATCGTTCAAGTGTGCGGCTAAATCCTTCAACGGTACGCAGTCAAATGCTAGAAAATGCATT
Ha_matK  TAAACCAATTATCCAATCATTCCCTTGACTTTCTGGGTTATCGTTCAAGTGTGCGTCTAAAGCCTTCAATGGTACGCGGTCAAATGCTAGAAAATACATT
Nt_matK  TAAACCAATTATCCAATCATTCCCGTGACTTTATGGGCTATCTTTCAAGTGTGCGACTAAATCCTTCAATGGTACGTAGTCAAATGTTAGAAAATTCATT
Cs_matK  TAAGTCAACTATCCAGCCATTCCCTTGACTTTATGGGCTATCTTTCAAGTATTCCACTAACCCCCTCAACGGTACGGAGTCAAATGCTAGAGAATTCTTT
At_matK  TAAATCAATTATCTAAAGATAATTTAGAGTTTCTGGGTTATCTGTCAAGTTTGCGATTAAACCCTTTAGTGGTACGTAGTCAAATGCTAGAAAACTCATT
         ***   ** **** *   **      ** *** **** ****  ****** * *   ***  ** * *  ******  ******** **** **  * **

Tco_matK TCTAATCGATAATGCTATTAAGAAGTTTGATACTCTTGTTCCAATTATGCCTCTGATTGGATCACTGGCTAAATCGAAATTTTGTAACGCATTGGGGCAT
Tci_matK TCTAATCGATAATGCTATTAAGAAGTTTGATACTCTTGTTCCAATTATGCCTCTGATTGGATCACTGGCTAAATCGAAATTTTGTAACGCATTGGGGCAT
Aa_matK  TCTAATCGATAATGCTATTAAGAAGTTTGATACTCTTGTTCCAATTATGCCTCTGATTGGATCACTGGCTAAATCAAAATTTTGTAACGCATTGGGGCAT
Ha_matK  TTTAATTAATAATGCTATTAAGAAGTTTGATAGTATTGTTCCAATTATGCCTCTGGTTGGATCGTTGGCTAAATCGAAATTTTGTAACGCATTGGGGCAT
Nt_matK  TCTAATCAATAATGCAATTAAGAAGTTCGATACCCTTGTTCCAATTATTCCTTTGATTGGATCATTAGCTAAAGCAAACTTTTGTACCGTATTAGGGCAT
Cs_matK  TTTAATAAATAATGCTATTAAGAAATTCGATACTATTGTTCCAATTATTCCTATCATTGGATCATTAGCTAAAGCTAAATTTTGTA-------ATGGAAT
At_matK  TCTAATAGATAATGTTAGAATCAAATTGGATAGCAAAATTCCAATTTCTTCTATTATTGGATCGTTGGCTAAAGATAAATTTTGTAATGTATTAGGGCAT
         * ****  ******  *  *  ** ** ****      ********    ** *  *******  * ******   ** *******         ** **

Tco_matK CCTATTGGCAAGGCGATTTGGACCGATTTATCAGATTCTGATATTATTGAGCGCTTTGGGCGTATATACAGAAATCTTTCTCATTATCATAGTGGATCTT
Tci_matK CCTATTGGCAAGGCGATTTGGACCGATTTATCAGATTCTGATATTATTGAGCGCTTTGGGCGTATATACAGAAATCTTTCTCATTATCATAGTGGATCTT
Aa_matK  CCTATTGGCAAGGCGATTTGGACCGATTTATCAGATTCTGATATTATTGAGCGCTTTGGGCGTATATACAGAAATCTTTCTCATTATCATAGTGGATCTT
Ha_matK  CCTATTGGTAAGGCAATTTGGGCCGATTTATCAGATTCTGATATTATTGAGCGCTTTGGCCGTATATACAGAAATCTTTCTCATTATCATAGTGGATCTT
Nt_matK  CCCATTAGTAAACCGGTTTGGTCCGATTTATCAGATTCTGATATTATTGACCGATTTGGGCGTATATGCAGAAATCTTTTTCATTATTATAGCGGATCTT
Cs_matK  CCCATTAGTAAACCGGTTTGGGCCGATTTATCAGATTCTGATATTATTGAACGGTTTGGGCGTATATACAGAAATTTTTTTCATTATTATAGTGGATCTT
At_matK  CCCATTAGTAAAGCAACCTGGACGGATTCATCAGATTCTGATATTCTCAACCGATTTGTGCGGATATGCAGAAATATTTCTCATTATTACAGCGGATCTT
         ** *** * **  *    *** * **** **************** *  * ** ****  ** **** ******* *** ******* * ** *******

Tco_matK CAAAAAAAAAGAGTTTGTATCGAGTAAAGTATATACTTAGACTTTCTTGTGCTAGAACTTTAGCTCGTAAGCATAAAAGTACTGTACGTGCTTTTTTGAA
Tci_matK CAAAAAAAAATAGTTTGTATCGAGTAAAGTATATACTTAGACTTTCTTGTGCTAGAACTTTAGCTCGTAAGCATAAAAGTACTGTACGTGCTTTTTTGAA
Aa_matK  CAAAAAAAAAGAGTTTGTATCGAGTAAAGTATATACTTCGACTTTCTTGTGCTAGAACTTTAGCTCGTAAGCATAAAAGTACTGTACGTGCTTTTTTGAA
Ha_matK  CAAAAAAAAAAAGTTTGTATCGAGTAAAATATATACTTCGACTTTCTTGTGCTAGAACTTTAGCTCGTAAGCATAAAAGTACTGTACGTGCTTTTTTGAA
Nt_matK  CCAAAAAAAAGACTTTATATCGAATAAAGTATATACTTCGACTTTCTTGTGCTAGAACTTTAGCTCGGAAACACAAAAGTACTGTACGCACTTTTTTGAA
Cs_matK  CCAAAAAAAGGA-----------------TATATACTTCGACTTTCTTGTGCTCGAACTTTAGCTCGTAAACATAAAAGTACTGTACGCACTTTTTTACA
At_matK  CAAAAAAAAAGAATTTGTATCGAATAAAATATATACTTCGTCTTTGTTGTGTTAAAACTTTGGCTCGTAAACACAAAAGTACTGTACGCACTTTTTTAAA
         * *******  *                 ********* * **** ***** *  ****** ***** ** ** *************   *******  *

Tco_matK AAGATTTGGTTCGGAATTATTGGAAGAATTCTTTATGGAAGAAGAACAAGTTTTTTCCTTGACCTTTCCAAGAGTTTCTTCTATTTCGCGAAGGTTATCT
Tci_matK AAGATTTGGTTCGGAATTATTGGAAGAATTCTTTATGGAAGAAGAACAAGTTTTTTCCTTGACCTTTCCAAGAGTTTCTTCTATTTCGCGAAGGTTATCT
Aa_matK  AAGATTTGGTTCGGAATTATTGGAAGAATTCTTTACGGAAGAAGAACAAGTTTTTTCCTTGACCTTTCCAAGAGTTTCTTCTATTTCGCGAAGGTTATCT
Ha_matK  AAGATTCGGTTCGCAATTATTGGAAGAGTTCTTTACGGAAGAAGAACAAGTTTTTTCCTTGACCTTTCCAAGAGTTTCTTCTATTTCGCGAAGGTTATCT
Nt_matK  AAGATCGGGCTCGGAATTATTGGAAGAATTCTTAACGTCGGAAGAACAAGTTCTTTCTTTGACCTTCCCACGAGCTTCTTCTAGTTTGTGGGGAGTATAT
Cs_matK  AAGCTTGGGTTCGGAATTCTTGGAAGAATTCTTTACGTCGGAAGAACAAATTCTTTCTTTCACTTTCCCAAACGCTTCTTCTACTTTGCGGGGAGTGTAT
At_matK  AAGGTTGGGCTCTGGTTTATTGGAAGAATTCCTTACGGGGGAAGACCAAGTTCTTTCTTTAATCTTCCCAAGAAGTTATTATGCTTCTAAAAGATTATAT
         *** *  ** **    ** ******** *** * * *   ***** *** ** **** ** *  ** ***     ** ** *  **      *  * * *

Tco_matK AGAAGGCGGATTTGGTATTTGGATATTATTTGTATCAATGATTTGGCCAATAATGAATGA
Tci_matK AGAAGACGGATTTGGTATTTGGATATTATTTGTATCAATGATTTGGCCAATAATGAATGA
Aa_matK  AGAAGGCGGATTTGGTATTTGGATATTATTTGTATCAATGATTTGGCCAATCATGAATGA
Ha_matK  AGAAGGCGGATTTGGTATTTGGATATTGTTTGTATCAATGATTTGGCCAATCATTAA---
Nt_matK  AGAAGTCGGATTTGGTATTTGGATATTTTTTGTATCAATGATCTGGCGAATTATCAATGA
Cs_matK  AAAAGGCGGATTTGGTATTTTGATATTGTTTGTATCAATGAGCTGGCCAATTATCAATGA
At_matK  CGAGTGCGAATTTGGTATTTGGATATTCTTTATCTTAATGATTTGGTCAATCATGAATAA
           *   ** *********** ****** *** * * *****  ***  *** ** **
```

Supplemental Figure S1.

Figure S1. Nucleic acid sequence alignments of internal transcribed spacer (ITS)1, ITS2, *rbcL* and *metK*. The sequences of internal transcribed spacer (ITS)1, and ITS2, ribu-lose-1,5-bisphosphate carboxylase/oxgenase large subunit (*rbcL*) and maturase K (*matK*) were obtained from the NCBI database or BLASTN. Accession No.; *T. cinerariifolium*: AB359720.1 (ITS1), AB359806.1 (ITS2) and MT104464.1 (*rbcL* and *matK*); *T. coccineum*: AB359721.1 (ITS1), AB359807.1 (ITS2) and MT104463.1 (*rbcL* and *matK*); *A. annua*: KC493085.1 (ITS1 and ITS2) and MF623173.1 (*rbcL* and *matK*); *H. annuus*: KF767534.1 (ITS1 and ITS2), L13929.1 (*rbcL*) and AY215805.1 (*matK*); *N. tabacum*: AJ300215.1 (ITS1 and ITS2), AP019625.1 (*rbcL*) and MZ707522.1 (*matK*); *A. thaliana*: X52320.1 (ITS1 and ITS2), NC_000932.1 (*rbcL*) and MK380721.1 (*matK*); *O. sativa*: KM036282.1 (ITS1 and ITS2), D00207.1 (*rbcL*) and KM103369.1 (*matK*). The sequences of ITS 1, ITS2, *rbcL* and *matK* of *C. seticuspe* were detected by BLASTN (version 2.7.1) with the sequences of these genes of *A. thaliana* as queries. The nucleic acid sequences (except *O. sativa* sequences due to their low similarity to other plants') were aligned using CLUSTAL W-mpi 0.13. Asterisks and hyphens denote conserved nucleic acids and gaps, respectively.

Supplemental Figure S2.

(A)

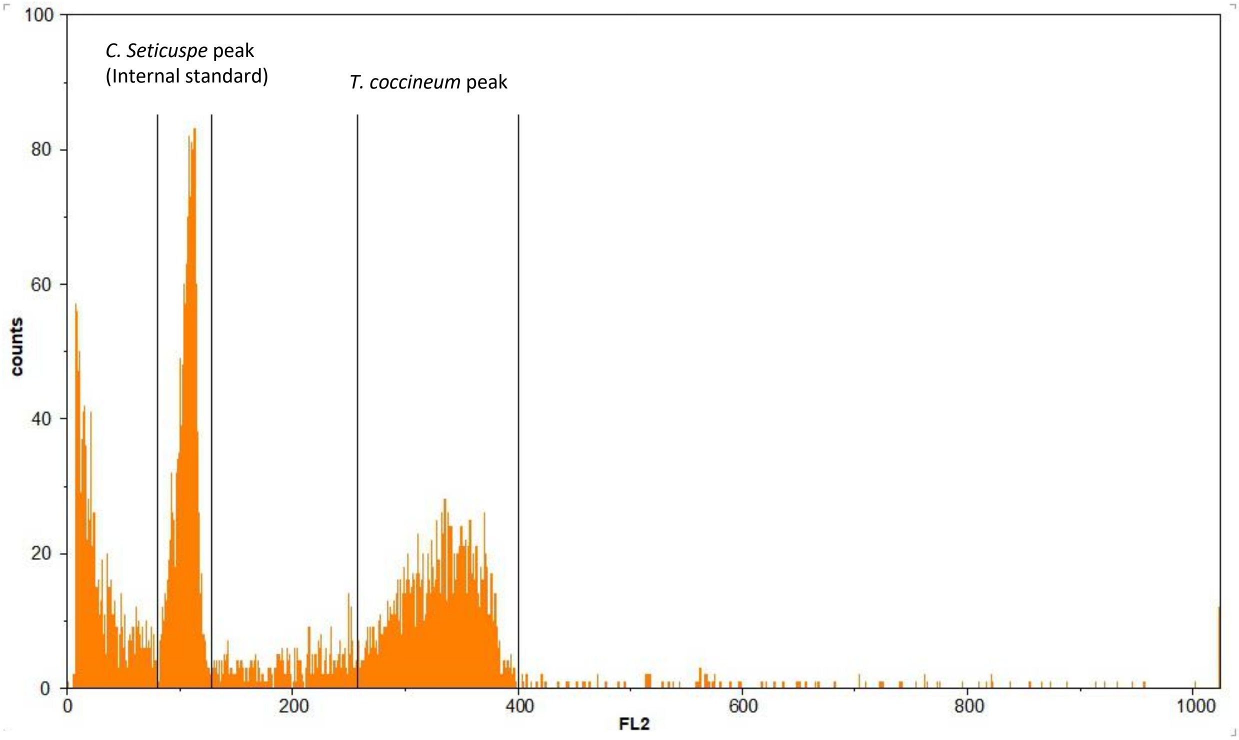

(B)

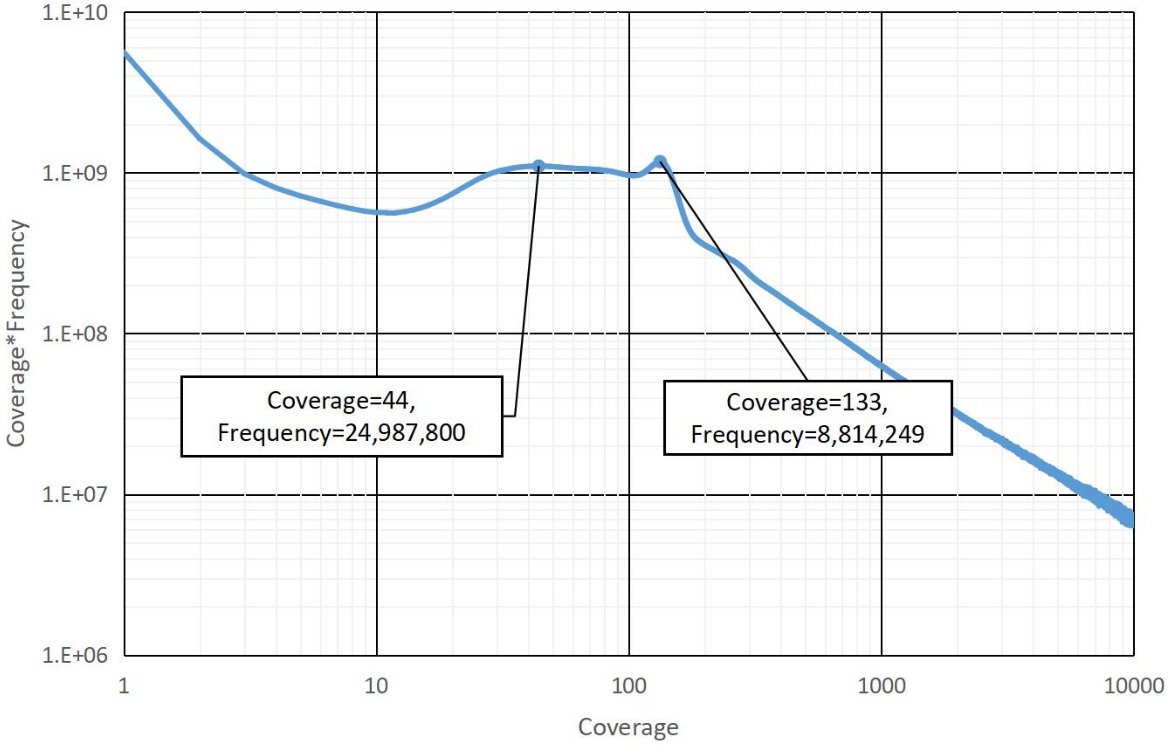

Figure S2. Size estimation of *T. coccineum* genome by flow cytometric analysis (A) and k-mer counting (B). (A) Estimation of relative nuclear DNA amount (genome size) of *T. coccineum*. The histogram of relative DNA content was obtained after flow cytometric analysis of propidium iodide-stained nuclei of *C. seticuspe* and *T. coccineum*. *C. seticupe* 'gojo-0' (3 pg/1C) served as internal reference standard. The peak of *C. seticuspe* was adjusted to be positioned near 100, with a final positioning at 106.10. The *T. coccineum* peak was positioned at 330.75; therefore, the 1C DNA amount of *T. coccineum* was estimated as 9.4 pg/1C. count: nuclei counts; FL2: relative DNA content. (B) k-mer spectrogram estimated from paired-end (PE) reads of *T. coccineum*.

Supplemental Figure S3.

```
Tco_0487905     MALNTPHVIIS--AVVHELGRSVMVEEIKVDPPKATEVRIKMLFASICHSDILCFDGFPTPLFPRIPGHEGVGMVESVGEDIKT
TcADH2          MSLNTPDVIICKAAVVRELGRSVMVEEIKVDPPKATEVRIKMLFASICHTDMLCFDGFPTPLFPRIPGHEGVGMVESVGEDIKT
                *:****.***.  ***:********************************:*:********************************

Tco_0487905     KLKPGDIVMPLLVGECGQCLNCKSGRTNLCLAYPLTLSGLMLDGTSRMSIAKTGETIYHHFSCSTWSEYMVIDINYVLKIDPKM
TciADH2         KLKPGDIVMPLFMGECGQCLNCKSKRTNLCHAYPLTLSGLLLDGTSRMSIAKTEETIYHHLSCSTWSEYMVIDINYVLKIDPKM
                ***********::*********** ***** *********:************ ******:***********************

Tco_0487905     HLPYASFLSCGFTTGFGASWKETQITEGSVVAVNGLGAVGLGAIKGAQMQGASKIIGIDVNENKAAKGKAFGMTHFINPKDHPN
TciADH2         HLPYASFLSCGFTTGFGAPWKETQITKGSIVAVFGLGAVGLGAIKGAQMQGASIIIGVDINENKAAKGKAFGMTHFINPKDHPN
                ******************.*******:**:*** ******************* ***:*:************************

Tco_0487905     QSVSDIVRDITYGLGVDYCFECTGISSLLKEIIEASKIGLGTTVLIGADADNIPIRSLSLINGRTLKGTTFGGVRTRSDLPIII
TciADH2         QLVSDMVRDITDGLGVDYCFECTGIASLLKEIIEASKIGFGTTILIGAAPDNVPISSLSLINGRTLKGTTFGGVRTRSDLPIIL
                * ***:***** *************:*************:***:**** .**:** ***************************:

Tco_0487905     QKCMNEVIELDELLTHEIRLENMHEVFEILKKPDCVKILINFA
TciADH2         QKCMNEEIELDELMSHEIRLENIHEIFEILKKPDCVKILINFD
                ****** ******::*******:**:****************
```

Red letters: NAD(P) binding cite

Blue bar: Alcohol dehydrogenase GroES-like domain (pfam08240)

```
Tco_0682217     MSSGANGNSKS-AYDIKFTKLFINGEFVDSISGSTFETIDPASEEVLATVAEGRKENVDLAVKAAREAFDNGPWPRLSGEARRK
TciALDH1        MSSGANGNSKSLAYDIKFTKLFINGEFVDSISGSTFETIDPATEEVLATVAEGREEDVDLAVKAAREAFDNGPWPRLSGEARRK
                *********** ******************************:***********:*:***************************

Tco_0682217     ILLKFADLIEENADEIATLEVIDTGKPFQIARYFENSWASETFRYFAGAADKIRGATLKMSSDFQAYTLREPIGVVGHIIPWNA
TciALDH1        ILLKFADLIEENADEIATLEVIDTGKPFQIARYVENSWTSETFRYFAGAADKIRGATLKMSSDFQAYTLREPIGVVGHIIPWNA
                *********************************.****:*********************************************

Tco_0682217     PAVLFAMKVAPALAAGCTMVIKPAENTPLGGLFMAYLSKLAGVPDGVINVVNGFGSTAGAAVSSHMDIDAVTFTGSTKVGRTIM
TciALDH1        PAYLFAMKVAPALAAGCTVVIKPAENTPLVGLFMAYLSKLAGVPDGVINVVNGFGSTAGAAVSSHMDIDAVTFTGSTKVGRTIM
                ** ***************:********** ******************************************************

Tco_0682217     QAAALSNLKPVSLELGGKSPFIVFDDADVDKAVEFAILGNLYNKGEMCIAGSRVFVQEGIHDVFVKKVEGALKAWATGDPFDLA
TciALDH1        QAAAASNLKPVSLELGGKSPFIVFDDADIEKAAEIAVLGVLSNKGELCVAGSRVFVHEGIYDAFVKKLEATVKNWATGDRFDAA
                **** ***********************::**.*:*:** * ****:*:*******:***:*.****:*.::* ***** ** *

Tco_0682217     TRHGPQNNKQQYEKVLSCIDHGKKEGATLVTGGKPFGKKGYYIEPTLFTNVTDDMTIAKEEIFGPVISVLKFKTVEEVIKRANA
TciALDH1        TRHGPQNNKQQYEKVLSYIELGKKEGATLVTGGKPFGNKGYYIEPTLFTNVTDEMTIAKEEIFGPVIMVLKFKTIEEVIRRANA
                ***************** *: ****************:***************:************* ******:****:****

Tco_0682217     TKYGLASGVFTKNIDVVNTVSRSIRAGAVWVNCYLALDRDAPHGGYKMSGFGREQGLEGLEHYLQIKTVATPIYDSPWL
TciALDH1        TTYGLAAGIMTKNIDIANTVTRSIRAGSVWVNCYLALDRDTPFGGYKMSGFGREQGLEALEHYLQVKTVTTPIYNSPWL
                *.****:*::*****:.***:******:************:*.***************.******:***:****:****
```

Red letters: NAD(P) binding cite

Blue bar: Aldehyde dehydrogenase family (pfam00171)

```
Tco_0360514     MVLFFLVLCLVILSYFLLNKRSVTGKTSLNLPPGPSKLPIIGNIYQLFGLPAHRAFRDLARKHGPIMHIQLGQISTVVISSPKL
TciCCH          MVLFFIVLCLIVLSYFLLNKRSVRGKTSLNLPPGPSKLPIIGNIYQLAGLPPHRAFRDLARKHGPIMHIQLGQISTVVISSPKL
                *****:****::*********** *********************** ***.********************************

Tco_0360514     AKEALKIHDIALADRPVTFTSDLVLYGNSDIALAPYGEYWRQMKKISSLELLSAKKVKSFGPIREQELDRFIDFLRLSSGKLVN
TciCCH          AKEALKIHDIALADRPVTFTSDLVLYGNSDIALAPYGEYWRQMKKISSLELLSAKKVKSFGPIREQELDYLIDFLRLSSGKLVN
                ********************************************************************* :*************

Tco_0360514     LHQTITKSINNIVCIASFGKNIKYQHELVEFLDVLAKTSSGFYVADLFPKFKLLYFVSGMRSKLLKLYRTLDKVFDDILKDHEQ
TciCCH          LHQTITKSINNIVCIASFGKNIKYQHELVEFLDMLARTNSGFYVADLFPKFKFLYFVSGMRSELLKLYRTLDKVFDDILKDHEQ
                *********************************:**:*.*************:*********:*********************

Tco_0360514     RR-IDKGDHVQEEDLLDVLLQVKNEGGLEFPITNNNIKAIFVDVFAGGTDTSSFTIEWAMTEMIRHPRVMKKAQDEVREAFKGK
TciCCH          RRIIDKGDHGQEEDLLDVLLQVKNKGGLEFPITNNNIKAIFVDIFAGGTDTSSNTIEWAMTEMIRHPRVMKKAQDEVREAFKGK
                ** ****** **************:******************:********* ******************************

Tco_0360514     TKIKETDIQCLTYLQSVIKETLRLHLPLPLLIPRVCREQCEIGGYDIPVKTKVIVNAFACSTDPEYWEDAETFKPERFDNSSVD
TciCCH          TKIKETDIQCLTYLPSVIKETLRLHVPLPLLIPRVCREQCKIGGYDIPVNTKVIVNAFACSTDPEYWEDAETFKPERFDNSSVD
                ************** **********:**************:********:**********************************

Tco_0360514     FIGSNYEFIPFGSGRRMCPGINFGLVSVKLFLAQMLYSFDWKLPDGLSPMELDMTEVESSIVAKKVNLQLIPTSYAPQ
TciCCH          FTGSNYEFIPFGSGRRMCPGINFGLVSVKLFLAQMLYYFDWKLPDGLSPRKLDMTEIESSIVAKKVNLQLIPTTYAPQ
                * *********************************** *********** :*****:****************:****
```

Red letters: heme binding site

Blue bar: Cytochrome P450 (pfam00067)

Supplemental Figure S3.

```
Tco_1190813     MTSQEPAAMEVADFLRMNTGNPESSYATNSSVQEIAIRKTEAVLKETIKSIANLHGFPQCFNIADLGCSSGPNTLLAISNIIHE
TciCCMT         MTSQEPAAMEVADFLRMNTGNPESSYATNSSVQEIAIRKTEAVLKETIKSIANLHGFPQCFNIADLGCSSGPNTLLAISNIIHE
                ************************************************************************************

Tco_1190813     VHEVCKEKNLKPPQLQVFLNDLFGNDFNSVFKSLPMFYANYNKEEGENNMCFVSAVPGSFHGRLFPDHSMHFFHSSTSLHWLSQ
TciCCMT         VHEVCKEKNLKPPQLQVFLNDLFGNDFNSVFKSLPMFYANYNKEEGENNLCCVSAVPGSFHGRLFPDHSMHFFHSSTSLHWLSQ
                *************************************************:* ********************************

Tco_1190813     VPKGIENNKLNICISETSPPNVFENYRMQFQKDFTTFLESRSIEIVHGGRMVLTFLGRGNVDPCCNGSGRVMELLGKSLVDAVN
TciCCMT         VPKGIENNKLNIYISETSPPNVFENYRMQFQKDFTTFLESRSIEIVHRGRMVLTFLGRGNVDPCSDGSGRVMELLGKSLVDAVN
                ************ ********************************** ****************.:******************

Tco_1190813     EGFVQESDLNSFNMPVYNPYKDEISETIHNQGSFSLDMLETFEVNLDPYDTNYEN-VKASGEPNHGEGAAKMLRAVVEPMFVAH
TciCCMT         EGFVQESDLNSFNMPVYRAYKDEISETIHNQGSFSLDMLETFEINLDPYDTNYENVLKASGEPNHGEGAAKTLRAVVEPMLVAH
                *****************..************************:*********** :************** ********:***

Tco_1190813     FGNSAMECVFKKLEERVDEQMAIEKTRFFFILISLTRI
TciCCMT         FGNSAMECVFKKLEERV----AIEKTRFFFILISLTRI
                *****************    *****************
```

: SAM dependent carboxyl methyltransferase (pfam03492)

```
Tco_1315810     MSRCLLCSLASKWASWGASSRPHPSVQPFVTRKNVVRYHKPTSESSYSPLTTTLSSHLDSQFMQVYETLKSELIHDPSFEFDDD
TciCDS          MSWCLLCGLSSKWASWDASSRPHPSVQPFVTRKNVVRYHKPTSESSYSTLTTTLSSNLDSQFMQVYETLKSELIHDSSFEFDDD
                ** ****.*:******.*******************************.*******:*******************.*******

Tco_1315810     SRQWVERMIDYTVPGGKMVRGYSVVDSYQLLKGEELTEDEAFLACALGWCTEWLQAFILVLDDVMDSSHTRRGQPCWFRLPEVG
TciCDS          SRQWVERMIDYTVPRGKMVRGYSVVDSYQLLKGEKLTEDGAFLVCALGWCTEWLQAFLLIHDDIMDGSHTRRGQPCWFRLPGVG
                ************** *******************:**** ***.*************:*: **:**.************** **

Tco_1315810     VVAINDGILLRNHVHRILKKYFQGKPYYVHLLDLFNETEFQTVSGEMIDMICRLAGQKDLSKYSMTLNRRIVQYKGSYYSCYLP
TciCDS          VVAINDGVLLRNHVHRILKKHFKGKAYYVHLLDLFNETEFQTISGQMIDTIARLAGQKDLSKYSMSLNSRIAQYKSSYYSCYLP
                *******:************:*:**.****************:**:*** *.*************:** **.***.********

Tco_1315810     IACALLMFGENLEDHVQVKDILVELGMYYQIQNDYLDTFGDPDVFGKTGTDIEECKCSWLIAKALELANEEQKKILSENYGIKD
TciCDS          IACALLMFGENLEDHVQVKDILVELGMYYQIQNDYLDTFGDPNVFVKTGTDIEECKCSWLIAKALELANEEQKKILSENYGIKD
                ******************************************:** **************************************

Tco_1315810     PEKVAKVKELYHALDLKGAYEDYETNLYEKSMTVIKAHPNIAVQAVLKTCLEKMYKGHK
TciCDS          PAKVAKVKELYHALDLKGAYEDYETNLYEKSMKAIKAHPSISVQAVLKSCLEKMYKGHK
                * ******************************..*****.*:******:**********
```

Red letters: catalytic residues

: Polyprenyl synthetase (pfam00348)

```
Tco_1108878     MAVASRKLGALVLVAVLCLSLPTGCLSSQQAAALFVFGDSCFDPGNNNHINTHVNFRANFWPYGQSYFSSPTGRFSDGRIIPDF
TciGLIP         MAVASRKLGALVLVAVLCLSLPTGCLSSQQAAALFIFGDSVFDPGNNNHINTHVNFKANFWPYGQSYFSSPTGRFSDGRIIPDF
                ***********************************:**** ***************:***************************

Tco_1108878     IAEYASLPFIPAYLEPNNDFTHGANFASAGAGALIDSHAGLAVGLQTQLRYFGDLVNHYRQNLGDIKSRQLLSDAVYLLSCGGN
TciGLIP         IAEYASLPIIPAYLEPNNDFTHGANFASAGAGALIASHAGLAVGLQTQLRYFGDLVDHYRQNLGDIKSRQLLSDAVYLFSCGGN
                ********:************************** ********************:*********************:*****

Tco_1108878     DYQSPYYPYTQEQYVDIVIGNMTNFIKGIYEKGGRKFGIVTVPHIGCWPGMRAKLPGNTCHTAADELTRLHNQAFAKRLEHLEK
TciGLIP         DYQSPYYPYTQEQYVDIVIGNMTNVIKGIYEKGGRKFGVVNVPLIGCWPGMRAKQPGNTCNTEVDELTRLHNQAFAKRLEQLEK
                ************************.*************:*.** ********** *****:* .****************:***

Tco_1108878     QLEGFMYAKFDLSTAISDRMKNPSKYGFKEGETACCGSGPFGGIYNCGRTKEFKLCDNVTEYFFFDPFHPNEVASRQFAEMFWD
TciGLIP         QLEGFVYAKFDLSTAILNRMKNPSKYGFKEGESACCGSGPFGGNYDCGRIKEFGLCDNATEYFFFDPFHPNELASRQFAEMFWD
                *****:********** :**************:********** *:*** *** ****.*************:***********

Tco_1108878     GDSMVIQPYNLKALFEGKPSTKFLPNDEL
TciGLIP         GDSMVTQPYNLKALFEGKPSTKYLPNDEL
                ***** ****************:******
```

Red letters: active site

: GDSL-like Lipase/Acylhydrolase (pfam00657)

Supplemental Figure S3.

```
Tco_0572988      -MIPMMASVFLYILLLFPILYLIYIILPKINKNRSSRLNPPGPLGLPFTGNLHQIDSSSLHTSLWNLSKSYGPIVFLRFGSIQS
TciJMH           MMIPTMASVFVYILLPFPILYLINLVW-KINKNRLNRLNPPGPLGLPFIGNLHQIDSSSLHTCLLNLSKSYGPILFLRFGSIPS
                 *** *****:**** ******* ::  ****** .************ *************.* *********:******* *:

Tco_0572988      VVVSSASLAKEVYKTQDVIFSSRPTSVSQRKFSYNGLDIVFSPYNDYWRDMRKIFTIHLLSSKRVQSSRYIREDEVSLAMKKIH
TciJMH           IVVSSASLAKEVYKTQDVIFSSRPCFVSHRKFSYNGLDVVFSPSNQYWRDMRKIVTIHLLSSKRVQSSRYMREEEVSLAMNKIH
                 ***********************  **:******* **:**** *:*********.***************:**:******:***

Tco_0572988      KLALSSKHINLTELMMNVTSTIVMRVGFGKTYEDGHERREIVRLLGELQSMITDFFVADLWPGLPFASFIDRLTGKTDRLEKCF
TciJMH           KLALSSEHINLTELMMNVTTTVVMRVGFGKRYEDGHERTEIVRLLGELQSMLADFFVADLWPGLPFASLIDRLIGKTARLENCF
                 ******:************:*:********* ******* ************::***************:**** *** ***:**

Tco_0572988      QDLDSFYQSLIDERLNTQNTNSHHDKEEQDILDILLQLKKDQVSNPNELTNNHIKAIITDVLVAGSDTSAATVVWAMTALMKNP
TciJMH           RDLDLFYQSLIDERLNAQNTNS-HDQEDKNIMDILLHLKKDQVSSPIKLTDNHIKAMLTDVLAAGSDTSAATVVWAMTALMKNP
                 :*** ************:*****  **:*:::*:****:*******.* :**:*****::****.********************

Tco_0572988      KVMRKAQEEVRTVAGKKGAIDENDLAQLIYLKAIVKEIMRLYPAAPLLVPRETKKDTILQGYEIKQKTLVHVNAFAIARDPESW
TciJMH           KVMRKAQEEVRTVGGKKGAIDENDLAQLIYLKAIIKEIMRLYPAAPLLVPRETTKDTILQGYEIKEKTLVHVNAFAIARDPESW
                 *************.********************:******************.***********:******************

Tco_0572988      ENPEEFLPERFLGSDIDFRGNDFELIPFGGGRRICPGITLGVLMAELLLANLIYLFDWKLPVGMKIEDIDYEAKPGFTMHKKND
TciJMH           ENPEEFIPERFLGSDIDFRGNDFELIPFGAGRRICPGITLGVIMAELLLANLIYLFDWKLPVGMKIEDIDYEAKPGFTMHKKNE
                 ******:*********************.************:*****************************************:

Tco_0572988      LCLLAGVYL
TciJMH           LCLLAGVYL
                 *********
```

Red letters: heme binding site

▬ : Cytochrome P450 (pfam00067)

```
Tco_0863779      ------------------------------------------------MVVAAISEDLVKLVRVEKEKPVTFKVRAVLTVRNKNKE
TciLOX1          MALAKQIMGASLMDQKTSVFGSNLCLNHVLVNKHRLRLRKTRKNGSMVVAAISEDLVKLXRVEKEKPVTFKVRAVLTVRNKNKE
                                                                 ************* ************************

Tco_0863779      DFFKDTIFRKIDAITDQIGWNVVIQLFSNDIDPRTRAAKKSNEAVLKDWSKKSNVKTERVNYTADIMVDSDFGIPGAITISNKH
TciLOX1          DFFKDTIFRKIDAITDQIGWNVVIQLFSNDIDPRTRAAKKSNEAVLKDWSKKSNVKTERVNYTADIMVDSDFGIPGAITISNKH
                 ************************************************************************************

Tco_0863779      QKEFFLETITIEGFACGPVHFPCNSWVQSTKDLPNPRIFFTNQPYLPDETPAGLKSLRYQELKDLRGDGTGVRKLSDRIYDYDV
TciLOX1          QKEFFLETITIEGFACGPVHFPCNSWVQSTKDLPNPRIFFTNQPYLPDETPVGLKSLRYQELKDLRGDGTGVRKLSDRIYDYDV
                 ***************************************************.********************************

Tco_0863779      YNDLGNPDRGNDFVRPTLGGEKIPYPRRCRTGRVPSDTDITAESRVEKPFPLYVPRDEQFEESKANAFSTGRLRAVLHNLLPSM
TciLOX1          YNDLGNPDRGNDFVRPTLGGEKIPYPRRCRTGRVPSDTDITAESRVEKPFPLYVPRDEQFEESKANAFSTGRLRAVLHNLLPSM
                 ************************************************************************************

Tco_0863779      VTSISKKHDFKGFSQIDSLYSEGVFLKLGLQDDLLKKLPLPNLVTRLQESSQGGGLLKYDTPKILSKDKFAWLRDDEFARQTIA
TciLOX1          VTSISKKNDFKGFSQIDSLYSEGVFLKLGLQDDLLKKLPLPNLVTRLHESSQGGGLLKYDTPKILSKDKFAWLRDDEFARQTIA
                 *******:***************************************:************************************

Tco_0863779      GMNPVSIEKLKVFPPVSQLDPEKHGPQESALREEHIVGFLDGMTVKQAIEEDKLFIIDYHDIYLPFLDRINALDGRKAYASRTI
TciLOX1          GVNPVSIEKLKVFPPVSQLDPEKHGPQESALREEHIVGFLDGRTVKQAIEEDKLFIIDYHDIYLPFLDRINALDGRKAYATRTI
                 *:**************************************** *************************************:***

Tco_0863779      FYLNPCGTLKPVAIELSLPQALPGSESKRVLTPPSDATGNWLWQLAKAHVCSNDAGAHQLVHHFLRTHAATEPFILAAHRQLSA
TciLOX1          FYLNPSGTLKPVAIELSLPQALPGSESKRVLTPPSDATSNWMWQLAKAHXCSNDAGAHQLVHHFLRTHAAIEPFILAAHRQLSA
                 *****.********************************.**:******* ******************** *************

Tco_0863779      MHPIYKLLDPHMRYTLEINQLARQNLINADGVIEACFTPGRYGMEISAAAYKNWRFDLEGLPADLIRRGMAVPDPSKPHGLKLV
TciLOX1          MHPIYKLLDPHMRYTLEINQLARQNLINADGVIEACFTPGRYGMEISASAYKNWRFDLEGLPADLIRRGMAVPDPSKPHGLKLV
                 ************************************************:***********************************

Tco_0863779      IEDYPYASDGLMIWEAIQNWVKTYVNHYYPDSAQVCNDRELQAWYAESINVGHADLRHKDWWPTLAGADDLTSVLTTIIWLASA
TciLOX1          MEDYPYASDGLMIWEAIQNWVKTYVNHYYPDSAQVCNDRELQAWYAESINVGHADLRHKDWWPTLAGADDLTSVLTTIIWLASA
                 :***********************************************************************************

Tco_0863779      QHAALNFGQYPYGGYIPNRPPLMRRLLPDVNDPEYLSFHDDPQKYFLSALPSLLQSTKYMAVVDTLSTHSPDEEYIGERQQTDT
TciLOX1          QHAALNFGQYPYGGYIPNRPPLMRRLLPDVNDPEYLSFHDDPQKYFLSALPSLLQSTKYMAVVDTLSTHSPDEEYIGERQQTDT
                 ************************************************************************************

Tco_0863779      WSGDAEIVEAFYAFSAEIQRIEKEIEKRNSDTSLKNRCGAGVLPYELLAPSSGPGATCRGVPNSISI
TciLOX1          WSGDAEIVEAFYAFSAEIQRIEKEIEKRNSDTSLKNRCGAGVLPYELLAPSSGPGATCRGVPNSISI
                 *******************************************************************
```

▬ : Lipoxygenase (pfam00305)

Supplemental Figure S3.

```
Tco_1240348    MALSLTTSIALATLLFFVYKFATRSKSTKNSLPEPWRLPIIGHVHHLIGTIPHRGVMDLARKYGSLMHLQLGEVSTIVVSSPKW
TciPYS         MALSLTTSIALATILFFVYKFATRSKSTKNSLPEPWRLPIIGHMHHLIGTIPHRGVMDLARKYGSLMHLQLGEVSTIVVSSPKW
               *************:*****************************:****************************************

Tco_1240348    AKEILTTYDITFANRPETLTGEIVFYHNTDIVFAPYGEHWRQLRKLCTLELLSVKKVKSFQSLREEECWNLVQEIKASGSGRLV
TciPYS         AKEILTTYDITFANRPETLTGEIVAYHNTDIVLAPYGEYWRQLRKLCTLELLSVKKVKSFQSLREEECWNLVQEIKASGSGRPV
               ************************ *******:*****:******************************************* *

Tco_1240348    NLSENIFKLIATILSRAAFGKGIKDQKEFTEIVKEILRQTGGFDVADIFPSKKFLHHLSGKRYRLTSIHKKLDNLINNLVAEHT
TciPYS         NLSENIFKLIATILSRAAFGKGIRDQKEFTEIVKEILRQTGGFDVADIFPSKKFLHHLSGKRARLTSIHTKLDNLINNLVAEHT
               ***********************:************************************** ******.**************

Tco_1240348    VKTSSKTNETLLDVLLRLKDSAEFPLTADNVKAVILDMFGAGTYTSSVTIEWAISELIKCPRAMEKVQVELRKALNGKERIQEE
TciPYS         VKTSSKTNETLLDVLLRLKDSAEFPLTADNVKAIILDMFGAGTDTSSATIEWAISELIKCPRAMEKVQVELRKALNGKERIHEE
               *********************************:********* ***.*********************************:**

Tco_1240348    DIQELSYLIMVIKETLRLHPPLPFILSRECRQPVNLAGYEIPNKTKLIVNVFAINRDPEYWKDAKNFIPERFENSSTTVMGAEY
TciPYS         DIQELSYLNLVIKETLRLHPPLPLVMPRECRQPVNLAGYDIPNKTKLIVNVFAINRDPEYWKDAETFIPERFENSSTTVMGAEY
               ******** :*************:::.************:************************:.******************

Tco_1240348    EYLPFGAGRRMCPGAALGLANVQLPLANILYHFNWKLPNGASYDQIDMTESFGATAQRKTELLLVPSF
TciPYS         EYLPFGAGRRMCPGAALGLANVQLPLANILYHFNWKLPNGASYDQIDMTESFGATVQRKTELLLVPSF
               *******************************************************.************
```

Red letters: heme binding site

: Cytochrome P450 (pfam00067)

Figure S3. Amino acid sequence alignment of proteins encoded by loci corresponding to known pyrethrin-related genes. The amino acid sequence alignment of *T. coccineum* proteins corresponding to the *T. cinerariifolium* proteins TciADH2 (accession No. AUQ44118.1), TciALDH1 (accession No. AUQ44119.1), TciCCH (accession No. AGO03787.1), TciCCMT (accession No. QCP80351.1), TciCDS (accession No. ADO17798.1), TciGLIP (accession No. AFJ04755.1), TciJMH (accession No. AXL93690.1), TciLOX1 (accession No. AGO03785.1) and TciPYS (accession No. AXL93709.1) are shown. These alignments are generated by using CLUSTAL W-mpi 0.13. While the *T. coccineum* Tco_0863779 protein appears to lack the N-terminal region corresponding to the TciLOX1 homolog, the absence of this sequence reflects the lack of the corresponding sequences at the end of the scaffold assembly. Asterisks, colons, dots and hyphens denote conserved amino acids, conserved substitutions, semi-conserved substitutions and gaps, respectively. Tci: *T. cinerariifolium*; Tco: *T. coccineum*; ADH2: alcohol dehydrogenase 2; ALDH1: aldehyde dehydrogenase 1; CCMT: 10-carboxychrysanthemic acid 10-methyltransferase; CDS: chrysanthemyl diphosphate synthase; CHH: chrysanthemol 10-hydroxylase; GLIP: GDSL (Gly-Asp-Ser-Leu motif) lipase; JMH: jasmone hydroxylase; LOX1: 13-lipoxygenase; PYS: pyrethrolone synthase.

Supplemental Figure S4.

```
SNA-I(O22415.1)           SYTNNAIDAQIIKMPVFR----GGGYEKVCSVVEVTRRISGWDGLCVDVRDGHYIDGNTVQLGPCGN-ECNQLWTF
Tci_399175(GEY27201.1)    -------KPTAIRMPVPVPVPVAVGADEQCPYGEPTTNIIGRDGQCMDVKENQYGNGNPIILFPCGNAQRNQLWTF
Tco_1336120               -------NPRAIRMPVPV----TVGANEQCPNGEPTTNIIGRDGQCVHVKDNQYNNGNSIILWACGNALRNQLWTF
                                 ..  *:***        * :: *.  * * .* * ** *:.*::.:* :**.: * .***   ******

SNA-I(O22415.1)           RTDGTIRWLGKCLTT-----SSSVMIYDCNTVPPEATKWVVSTDGTITNPRSGLVLTAPQAAEGTALSLENNIHAA
Tci_399175(GEY27201.1)    KSDGTIRSNGKCLTT----SGNYIMIFDCD-LAPETTKWILHNAGTIMNPRLRLVIAAESSTPRTVLTAAVDSNSS
Tco_1336120               KSDGTIRSNGKCLTTFGYASGNYIMIFDCDTAVPEATKWFLYNAGTIMNPRSGLVIAAETSTQGTVLTVAKDNNSS
                          ::*****  ******     .. :**:**:   **:***.: . *** ***  **::*  ::  *.*:   : :::

SNA-I(O22415.1)           RQGWTVG-DVEPLVTFIVGYKQMCLTENGENNFVWLEDCVLNR-VEQEWALYGDGTIRVNSNRSLCVTSEDHEPSD
Tci_399175(GEY27201.1)    RQAWSAGNYTQPTITYISGFLEMCLQANGENARVWLANCVIDTEPRQQWAIYGDRTIRLYSDRTLCVTSDGHESVD
Tco_1336120               RQAWSAGNYTQPTINYISGYREMCLQANGANARVWLANCVIGTEPRQQWVLYGDSTIRLYSDRTLCVTSDGHESSD
                          **.*:.*  .:* :.:* *: :***  ** *  *** :**:.   .*:*.:*** ***: *:*:*****:.**. *

SNA-I(O22415.1)           LIVILKCEGSGNQRWVFNTNGTISNPNAKLVMDVAQSNVSLRKIILYPPTGNPNQQWITTTQPA
Tci_399175(GEY27201.1)    SIILFKCQGSEAQRWTFMADATILNPYAQLVMDVRGSDVSLQEIILYPPTGNPNQKWLAF----
Tco_1336120               SIILLKCQGWGDQRWTFMADGTILNPNARLVMDVRNSDVSLQEIILYQPTGNPNQNWLAF----
                           *:::**:*   ***.* ::.** ** *:*****  *:***::**** *******:*::
```

Figure S4. Amino acid sequence alignment of RICIN domain of ribosome-inactivating proteins. The amino acid sequence alignment of *Sambucus nigra* agglutinin I (SNA-I, accession No. O22415.1), *T. cinerariifolium* Tci_399175 (accession No. GEY27201.1), and *T. coccineum* Tco_1336120 is shown. This alignment is generated by using CLUSTAL W-mpi 0.13. RICIN domains are indicated by green bars. Conserved Q-X-W motifs in RICIN domain are indicated by red letters. Asterisks, colons, dots and hyphens denote conserved amino acids, conserved substitutions, semi-conserved substitutions and gaps, respectively.
